# Particle size distributions of wildfire aerosols in the western USA†

**DOI:** 10.1039/d5ea00007f

**Published:** 2025-03-13

**Authors:** Siying Lu, Chiranjivi Bhattarai, Vera Samburova, Andrey Khlystov

**Affiliations:** a Division of Atmospheric Sciences, Desert Research Institute Reno Nevada USA andrey.khlystov@dri.edu; b Department of Physics, University of Nevada Reno Nevada USA

## Abstract

Wildfires are a major source of aerosols during summer in the western United States. Aerosols emitted from wildfires could significantly affect air quality, human health, and the global climate. This study conducted a comparison of aerosol characteristics during wildfire smoke-influenced and non-smoke-influenced days. Ambient particle size distribution (PSD) data were collected in Reno, Nevada, between July 2017 and October 2020. During this period, the site was impacted by smoke from over a hundred wildfires burning in a wide range of ecosystems in the western United States located at different distances from the measurement site. The smoke-influenced days were identified using satellite images, a hazard mapping system, and wind back-trajectory. Positive matrix factorization (PMF) was applied to identify the main sources and their characteristics. The wildfire aerosols were observed to have a number mode diameter of 212 nm, which is significantly larger than aerosols on non-smoke-influenced days (61 nm). In addition to the increase in particle size, wildfires made a large contribution to PM_2.5_ and CO concentrations. During fire-prone months (July, August, and September) from 2016 to 2021, 56% to 65% of PM_2.5_ and 18% to 26% of CO concentrations could be attributed to wildfire emissions in the study area. On an annual basis, wildfire emissions were responsible for 35% to 47% of PM_2.5_ concentrations and 5% to 12% of CO concentrations.

## Abstract 2

Wildfires are a major source of aerosols during summer in the western United States. Wildfire aerosols tend to be significantly larger than non-fire ambient aerosols.

## Graphical abstract

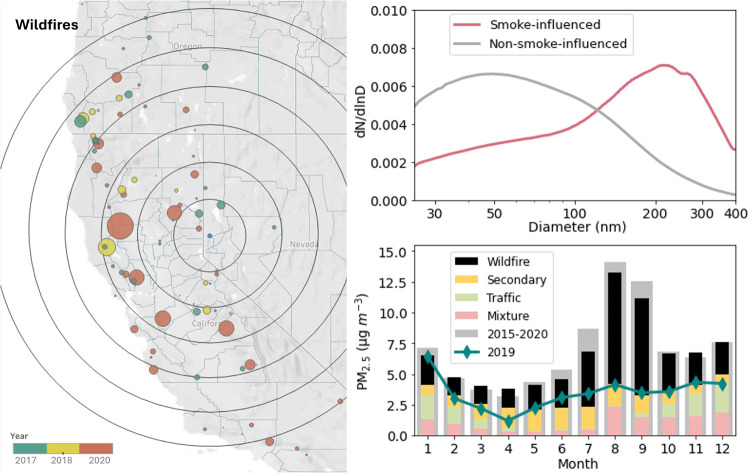

**Environmental significance**

As wildfire frequency and intensity continue to increase, characterizing the particle size distribution of wildfire emissions becomes increasingly vital for accurately assessing the climate effects of wildfire aerosols and evaluating potential health risks associated with smoke exposure. This study investigated aerosol particle size distributions measured in Reno, Nevada, over a 16 months period, during which the measurement site was impacted by smoke from 106 wildfires burning in a wide range of ecosystems in the western United States located at different distances from the measurement site. We show that wildfire-related aerosols are considerably larger (a number mode diameter of 212 nm) than aerosols on non-smoke-influenced days (61 nm). In addition to the increase in particle size, wildfires made a large contribution to local air pollutant concentrations. For example, wildfires contributed 35% to 47% of PM_2.5_ on an annual basis. The findings will enhance our ability to model and predict both the climatic and health impacts of wildfire emissions, supporting more effective air quality management strategies and public health interventions in regions affected by wildfire smoke.

## 1. Introduction

Atmospheric aerosols influence the global radiation balance by directly scattering and absorbing solar radiation.^1–3^ They also affect cloud formation^4^ and albedo.^5^ As one of the six criteria air pollutants,^6^ particulate matter (PM) directly affects air quality,^7–9^ visibility,^10^ and health.^11,12^

Biomass burning (BB), which includes wildfires, is a major source of atmospheric aerosols.^13^ The frequency, size, and severity of wildfires in the western United States have increased over the past two decades.^14–16^ Studies worldwide – such as those from the United States,^17^ Finland,^18,19^ Australia,^20^ Brazil,^21^ Mexico,^22^ China,^23^ and Russia^24^ – have shown that fires can generate large amounts of aerosols, raising concerns about their impact on air quality, human health, and climate. Wildfire emissions have been linked to adverse health effects,^12,25,26^ such as cardiorespiratory diseases,^27^ asthma attacks,^27,28^ and overall respiratory morbidity.^28,29^ Wildfire-emitted aerosols also influence the global and regional climate by scattering and absorbing solar radiation, and impacting clouds and precipitation.^30,31^ It has been demonstrated that wildfire emissions are associated with changes in atmospheric circulation,^9,30^ increases in Arctic sea ice,^30^ and other climate anomalies such as droughts.^31^ However, there is still significant uncertainty in the current estimates of wildfire effects on local and global climates.^32–34^

Aerosol particle size distribution (PSD) plays a significant role in how aerosols affect the climate^35,36^ and human health.^37^ For example, direct radiative forcing caused by wildfire aerosols is sensitive to aerosol PSD.^38^ Fine particles are particularly important in total optical extinction because of their high mass scattering efficiency and absorption cross-sections.^39^ The ability of particles to act as cloud condensation nuclei (CCN) is not only driven by their chemical composition, but also primarily by their size.^40^ BB aerosols can significantly elevate CCN numbers.^41^ Zheng *et al.*^42^ demonstrated that CCN concentration in the remote marine boundary layer could be enhanced by long-range transported wildfires. Aerosol size is also a key controlling parameter of aerosol deposition in the human respiratory tract,^37,43^ which influences exposure to toxic aerosol components and the resulting negative health effects. Therefore, PSD measurements of wildfire-emitted aerosols are needed to understand the effects of these aerosols on the climate and human health.

Several studies have reported PSD measurements of wildfire aerosols. Chubarova *et al.*^24^ detected an increase in the volume PSD mode radius from 0.15 μm under typical conditions to 0.24 μm during a fire event in Russia. McMeeking *et al.*^44^ observed that the volume geometric mean diameter (GMD) in Yosemite National Park, CA, was elevated by 0.06 μm during wildfire episodes compared to non-smoke periods. Veselovskii *et al.*^17^ recorded a volume PSD radius of approximately 0.27 μm in the summer, which was influenced by forest fires near Washington DC. Kleinman *et al.*^45^ demonstrated that particle size increases with downwind distance, potentially enhancing the cooling effect in the western United States. In addition, June *et al.*^46^ reported that smoke with higher initial organic aerosol concentration exhibits faster particle growth compared to smoke with lower initial organic aerosol concentration. Using a scanning mobility particle sizer (SMPS), Zheng *et al.*^42^ demonstrated a number PSD mode of aged wildfire aerosols at a diameter of 230 nm. Laing *et al.*^47^ reported that aerosols originating from wildfires in the western United States had a number GMD ranging from 138 to 229 nm. In Southern California, the number PSD mode during fire episodes were significantly larger, with diameters between 0.1 and 0.2 μm, compared to typical urban air conditions.^48^ Okoshi *et al.*^49^ demonstrated a number PSD mode at a diameter of ∼100 nm during small wildfire events and suggested using size measurements over mass measurements for ambient wildfire events. Studies of wildfire smoke reporting size distribution measurements covering a wide particle size range are limited with notable variations in the reported GMD or mode sizes. The underlying causes of these variations – such as differences in measurement methods, wildfire characteristics, and distances – remain uncertain.

The aim of this study was to examine the effect of wildfires on the concentration and PSD of atmospheric aerosols in the western United States using PSD data measured in the 25 to 400 nm diameter range in Reno, NV, between July 2017 and October 2020. During this period, the study area was impacted by 106 wildfires burning in a wide range of ecosystems and located at distances ranging from 24 to 700 km from the measurement site. The dataset therefore provides a robust sample of wildfire aerosols that could be encountered in the western United States. Positive matrix factorization (PMF) analysis was performed to better understand the contribution of wildfire to the ambient aerosol PSD and air quality relative to other air pollution sources.

## 2. Measurements and methods

### 2.1 Location

**Fig. 1:**
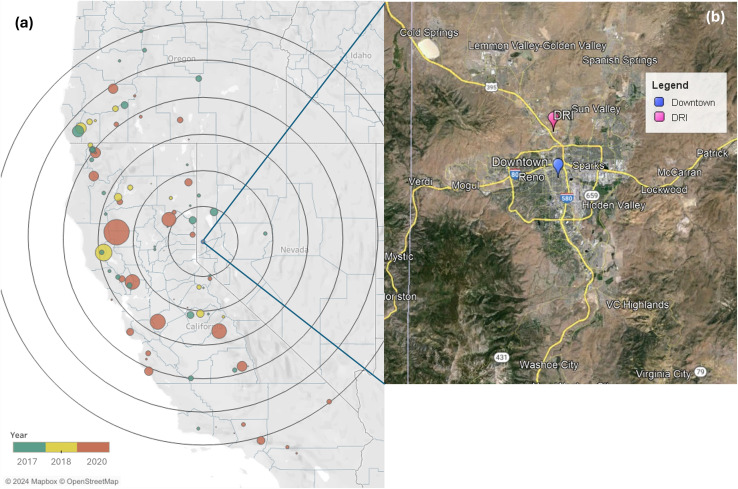
The left panel (a) shows a geospatial representation of wildfires that were affecting Reno, NV, during the observation period. The size of each wildfire dot represents the burned area (km^2^). Concentric black circles radiate from Reno, NV, with each circle representing a 100 km increment from the city, which serves as a spatial reference for distance. The right panel (b) shows a detailed map of the Reno area, including downtown Reno and DRI, which is adapted from Google Earth.

PSD measurements were carried out on the roof of the Desert Research Institute (DRI) building located at 39°34′20.67′′ N 119°48′06.83′′ W, in northern Reno, NV, from July 2017 to August 2018, April 2020 to May 2020, and August 2020 to October 2020. Fig. 1a shows a map depicting the locations of the measurement site and 106 fires whose smoke could have impacted the area during the study period (see Section 2.4). Most of these wildfires occurred in California, with additional events in Nevada and Oregon. Table S1† provides information on each wildfire, including the start date, end date, burned area, location, and type of vegetation burned.

Reno is a city located on the east side of the Sierra Nevada and the west of the Great Basin (Fig. 1b) with an area of approximately 289 km^2^ and a population of 274 915 people in 2023.^50^ The elevation of this city is approximately 1300 meters above sea level. Reno has a semiarid climate with low annual precipitation (average annual accumulated precipitation from 1991 to 2020 was 187 mm according to U.S. Climate Data^51^). The main local sources of PM in Reno are traffic and domestic burning.^52^ Aerosols can also be transported from neighboring states such as California and Oregon.^53–55^

### 2.2 Instrumentation

The aerosols were measured with an SMPS that consists of a differential mobility analyzer (DMA) model 3081 from TSI (St Paul, MN, USA) and a condensation particle counter (CPC) model 3775 from TSI. The PSD was measured with 5 minutes upscans and 30 seconds downscans (a total of 330 seconds per one size distribution measurement) in a particle diameter range of 25 to 400 nm.

Hourly and daily air pollutant concentrations of PM_2.5_ (aerosol particles with aerodynamic diameters of or less than 2.5 μm), ozone (O_3_), nitric oxide (NO), nitrogen dioxide (NO_2_), nitrogen oxides (NO_*X*_), carbon monoxide (CO), potassium (K), elemental carbon (EC), and organic carbon (OC) measured at the downtown Reno air quality monitoring site (Fig. 1, Environmental Protection Agency (EPA) Air Quality System identification no. 320310016 in 2017 and 2018, no. 320310031 in 2020) were downloaded from the EPA website.^56^

The wind back-trajectory data were processed using the National Oceanic and Atmospheric Administration (NOAA) Hybrid Single-Particle Lagrangian Integrated Trajectory (HYSPLIT) Model.^57,58^

Satellite images were taken from Worldview, the National Aeronautics and Space Administration (NASA) with the Fires and Thermal Anomalies (day and night) layer, which shows active fire detections and thermal anomalies, from MODIS Terra (MODIS/Terra Thermal Anomalies/Fire 5-Min L2 Swath 1 km, MOD14) and Aqua (MODIS/Aqua Thermal Anomalies/Fire 5-Min L2 Swath 1 km, MYD14) satellite product with 1 km sensor resolution and daily temporal resolution.

The daily smoke polygon product from the NOAA Hazard Mapping System Fire and Smoke Product (HMS) was used for additional identification of the presence of smoke. The HMS with 2 km nominal spatial resolution is based on two satellite products (GOES-16 and GOES-17). Ecoregions and their vegetation features for California, Oregon, and Nevada were collected from ecoregion posters on the EPA Ecoregions website.^59^

### 2.3 Data processing

The SMPS data were averaged to hourly values from which the aerosol number concentration (units: cm^−3^), volume concentration (units: μm^3^ cm^−3^), and the number GMD (units: nm, hereafter referred to simply as GMD) were calculated. The total number concentration was calculated by summing up the number concentration of particles over the 25 to 400 nm size bins. To get the volume concentration, the number concentration for different particle size bins was multiplied by 
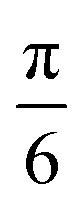
 and the cube of particle diameters, and then summed up. The following equation was used to calculate GMD:1
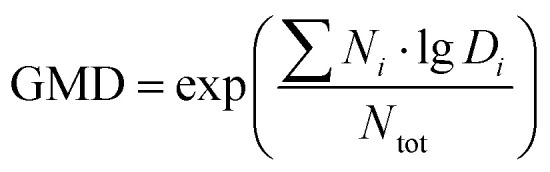
where *N*_*i*_ is the number concentration of particles with different diameters at different datetimes, *D*_*i*_ is the particle diameter, and *N*_tot_ is the total number concentration for all particles at different diameters.

To gain insight into prevailing sources and whether they correlate with certain PSD characteristics, positive matrix factorization (PMF) implemented with the Python program scikit-learn package^60^ was applied to the PSD hourly data. PMF has been used extensively in ambient air quality studies.^61–66^ The PMF can be represented as:^67–69^

2 *X* = *GF* + *E*

where *X* (*n* × *m*) is the matrix of input data with dimension *n* rows and *m* columns, *G* (*n* × *p*) is the contribution/weight matrix where *p* is the number of factors, *F* (*p* × *m*) is the matrix of factor profile, and *E* (*n* × *m*) is the residual matrix.

The EPA PMF 5.0 program was used to quantify the contribution of air pollutant sources for an 8 year (2015 to 2022) data analysis using daily air pollutant concentrations. The program requires chemical species concentrations with uncertainties as input data and produces factor contributions, factor profiles, and residuals. The best run was selected based on the critical goodness-of-fit parameter *Q*, with the lowest value indicating the optimal choice. Bootstrap (BS), displacement (DISP), and BS-DISP error estimation analysis methods were used to estimate the variability of the selected run and to determine the optimal number of factors. A Student's *t*-test was conducted to determine the statistical significance of the differences between the means of two groups. The significance level (*p*-value) was set at 0.05.

### 2.4 Identification of smoke-influenced days

**Fig. 2:**
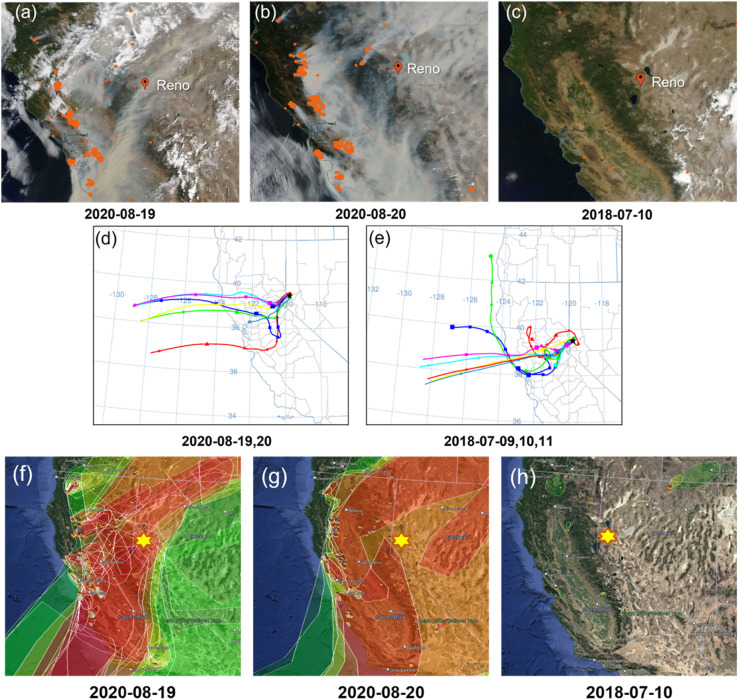
Satellite images during smoke-influenced days of (a) August 19, 2020, and (b) August 20, 2020, as well as a baseline day (c) of July 10, 2018. The small orange dots in (a)–(c) are fire and thermal anomaly spots that could have affected the Reno, NV, area. The wind back-trajectories for Reno during the smoke-influenced days (d) of August 19 and 20, 2020, and the baseline days (e) of July 9, 10, and 11, 2018, were produced at heights of 100 m, 500 m, and 1000 m starting a new trajectory every 6 hours. The HMS results during smoke-influenced days (f) of August 19, 2020, and (g) August 20, 2020, and a baseline day (h) of July 10, 2018. In (f), (g), and (h), the shadows with different colors indicate different smoke levels (green = light; yellow = medium; red = heavy). Reno, NV, is marked by a red marker in (a)–(c), a black star in (d) and (e), and a yellow hexagram in (f)–(h).

Satellite images, wind directions, and wind back-trajectories were investigated to verify that the measurement site was indeed impacted by a wildfire and to identify the fire location. Satellite images were used to observe whether smoke was affecting the area around the site. The back-trajectories were then used in conjunction with local wind directions and areas of satellite thermal anomalies to locate fires that might affect the measurement site. Fig. 2 shows examples of using satellite imagery and wind back-trajectories to identify smoke-influenced days and the fires that could have affected the measurement site. During August 19 and 20, 2020, the wind back-trajectories and the western and southwestern winds indicated that smoke generated by wildfires could have been transported to the Reno area (Fig. 2a and b). In contrast, during the non-smoke-influenced days, when the site was again subject to the westerly airflow (Fig. 2e), there were no major fires in the upwind direction, whereas the absence of smoke on the satellite images further confirmed the absence of influence from wildfire smoke (Fig. 2c). Along with satellite images and wind back-trajectory plots, HMS maps were used for additional confirmation of smoke-influenced days by checking the presence of overhead smoke in Reno, NV, during 2018 to 2020 (HMS is not available for 2017). HMS indicated medium to heavy smoke over Reno, NV, on August 19 and 20, 2020 (Fig. 2f and g), whereas no smoke was indicated on July 10, 2018 (Fig. 2h). This confirms that August 19 and 20, 2020, were smoke-influenced days, whereas July 10, 2018, was not. In addition, the HMS helped to exclude September 1, 2020, when fires and smoke were around Reno, but did not affect the city area. However, the HMS appeared to miss some smoke-influenced days. For example, HMS did not indicate the smoke impact on September 26, 2020, in the Reno area, but our analysis of satellite images and wind back-trajectories indicated that the site was impacted by fires, which was further confirmed by a high PM_2.5_ concentration (reaching 58 μg m^−3^) and a high total aerosol volume concentration (reaching 31 μm^3^ cm^−3^), which are typically in the range of 3 to 13 and 0 to 9 during non-smoke-influenced days, respectively.

The largest uncertainty in our approach in smoke-influence day identification comes from the use of satellite imagery, which provides only one snapshot per day. Thus, there is a chance that smoke influence could be missed if it occurred at a different time of day. Another uncertainty could arise from the fact that the satellite measurements provide a column-average information. If a smoke plume is transported at higher levels without affecting the ground, the image would provide a false positive indication for our ground-based measurements. However, with a few exceptions, our smoke identification correlated well with the observed differences in aerosol PSD and pollutant concentrations, as will be shown in the Results and discussion section.

In total, for the period of July 2017 to October 2020, 88 days were identified as smoke-influenced days (July 14, 16, 19–20, 25, and 30–31, 2017; August 1–5, 7, and 29–31, 2017; September 1–3, and 14–15, 2017; October 11–12, 2017; July 4, 14–15, 20–22, and 27–31, 2018, August 1–12 and 16, 2018; August 16–31, 2020; September 2–8, 10–22, 26, and 30, 2020; October 1 and 4–5, 2020). Seven days (from July 5 to 11, 2018) were used as a baseline for comparison with smoke-influenced days. These baseline days were selected in smoke-influenced months (mainly July, August, and September) in Reno, NV,^70^ to reduce the influence of seasonal variation in aerosol sources. Days that were not selected as the smoke-influenced days or the baseline days were assigned as “other” days.

## 3. Results and discussion

### 3.1 Comparison of smoke-influenced and non-smoke-influenced days

**Fig. 3:**
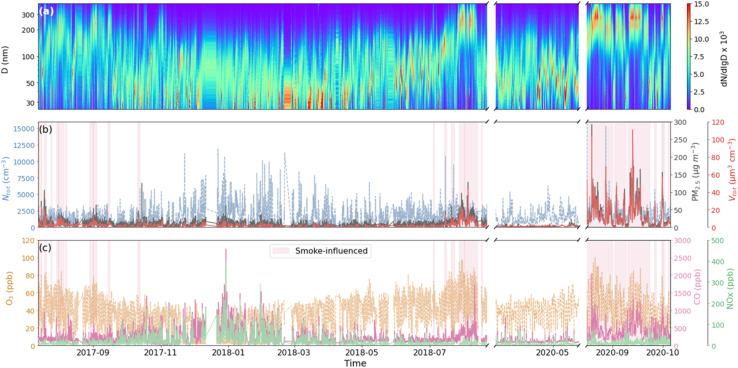
Time-resolved (a) normalized number PSD distribution with the log-normal fit particle diameter; (b) *N*_tot_, *V*_tot_, and PM_2.5_ concentration; and (c) O_3_, CO, and NO_*X*_ concentration. The red shadow areas represent the smoke-influenced days.

Fig. 3 shows a time series of the number PSD, total number concentrations (*N*_tot_), and total volume concentrations (*V*_tot_) measured with the SMPS at DRI as well as air pollutant concentrations (PM_2.5_, O_3_, CO, and NO_*X*_) measured in downtown Reno, NV, during the period when the SMPS measurements were done. The periods identified as smoke-influenced are shaded in red.

Particle sizes, *V*_tot_, and PM_2.5_ concentrations during smoke-influenced days tend to be significantly higher than average. Steep increases in *V*_tot_ and PM_2.5_ concentration were observed during the smoke-influenced days, with the highest *V*_tot_ exceeding 135 μm^3^ cm^−3^, and PM_2.5_ concentration reaching 292 μg m^−3^. Therefore, *V*_tot_ and PM_2.5_ concentrations can be used to confirm fire influence. CO concentrations had significant increases during both winter and the smoke-influenced days, which is reasonably following the emissions from traffic and wildfires. On the other hand, *N*_tot_, O_3_, and NO_*X*_ concentrations did not provide a clear indicator for the smoke-influenced days. High values of O_3_ concentrations are regularly observed because of the seasonal photochemical pattern. The elevated winter concentrations of particle number, NO_*X*_, and CO were likely due to lower atmospheric mixing and additional emissions from domestic wood burning.

**Fig. 4:**
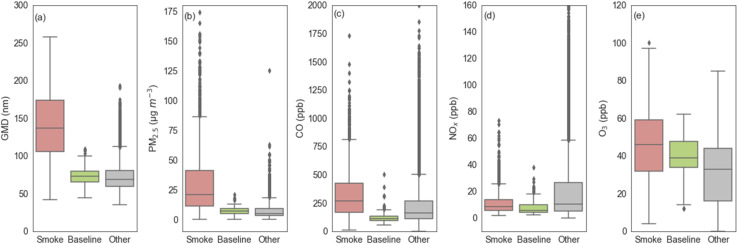
Boxplots of hourly (a) GMD, (b) PM_2.5_ concentration, (c) CO concentration, (d) NO_*X*_ concentration, and (e) O_3_ concentration during the smoke-influenced days (pink on the left), the baseline days in fire month (green in the middle), and the other days (gray on the right).

Fig. 4 shows boxplots of GMD and air pollutant (PM_2.5_, CO, NO_*X*_, O_3_) concentrations measured during the identified smoke-influenced days, the baseline days, and the other days. Their descriptive statistics are provided in Table 1. GMD and PM_2.5_ concentration had considerably higher values during smoke-influenced days than during non-smoke-influenced days (baseline + other) with high statistical significance (*p* < 0.001). GMD during smoke-influenced days (approximately 139 nm on average) was approximately two times greater than GMD during baseline days and other days. Of the smoke-influenced days, August 19, 2020, had the largest GMD of approximately 257 nm and September 1, 2020, had the smallest GMD of approximately 42 nm. In contrast, the largest GMD during the baseline days was approximately 109 nm on July 7, 2018, and the smallest GMD was 44 nm on July 9, 2018. This shows that the smallest GMD observed during smoke-influenced and non-smoke-influenced days were similar, but most of the observed GMD during the smoke-influenced days were significantly (more than two times) larger. Portin *et al.*^71^ showed similar results during summer: higher GMDs during smoke-influenced days of 158 nm compared to the mean sizes on the other days of 76.3 nm. However, Alonso-Blanco *et al.*^72^ found smaller particle sizes during days with wildfires than days without fires, although their measurements were limited to the size range above 0.1 μm. The similarity of the smallest GMD between smoke-influenced and non-smoke-influenced days observed in this study is likely due to some periods during the smoke-influenced days having little or no fire impact. Our smoke-influenced/non-smoke-influenced designation is mostly based on daily satellite observations, whereas size distributions were measured at a much higher frequency. The smoke-influenced days may include periods of low fire smoke impact. The correlation of the observed GMD with the other pollutants will be discussed later in the paper.

**Table 1:** Descriptive statistics of hourly mean data measured during 2017, 2018, and 2020

	Type of day	Number of data points	Mean	Std	Min	25%	50%	75%	Max
GMD (nm)	Smoke	2097	139	45.2	42.0	106	136	174	257
	Baseline	168	72.3	12.2	44.1	65.1	72.6	79.3	109
	Other	8630	72.2	19.4	35.4	59.1	69.6	80.3	193
PM_2.5_ (μg m^−3^)	Smoke	1949	31.0	29.2	0.00	11.0	21.0	41.0	292
	Baseline	143	7.27	3.43	0.00	5.00	7.00	9.00	21.0
	Other	19 227	6.70	5.63	0.00	3.00	5.00	9.00	125
CO (ppb)	Smoke	1949	329	222	11.0	166	266	424	2036
	Baseline	143	126	59.5	57.0	94.0	112	133	504
	Other	19 227	231	206	1.00	112	159	269	2747
NO_*x*_ (ppb)	Smoke	1949	10.9	8.35	1.90	5.40	8.40	13.50	73.2
	Baseline	143	8.00	6.02	2.20	4.15	5.70	10.0	38.2
	Other	19 227	21.1	26.2	0.10	5.40	10.50	26.7	395
O_3_ (ppb)	Smoke	1949	45.6	18.3	4.00	32.0	46.0	59.0	100
	Baseline	143	39.4	11.6	12.0	34.0	39.0	47.5	62.0
	Other	19 227	30.6	17.8	0.00	16.0	33.0	44.0	85.0

The mean and median values of PM_2.5_ concentration during baseline and other days were approximately 1/4 to 1/3 of the values during smoke-influenced days. The maximum value of PM_2.5_ concentration during smoke-influenced days reached 292 μg m^−3^, which was more than 13 times the maximum value during the baseline days and more than 2 times the maximum value during the other days. This confirms that in the western United States, wildfire is a significant source of PM_2.5_ in summer.^73^ CO had higher concentrations during smoke-influenced days than non-smoke-influenced days as well, but not as pronounced as the differences in the GMD and PM_2.5_ concentrations. NO_*X*_ and O_3_ concentrations had comparable values during smoke-influenced and baseline days, which were both lower than their values during the other days.

**Fig. 5:**
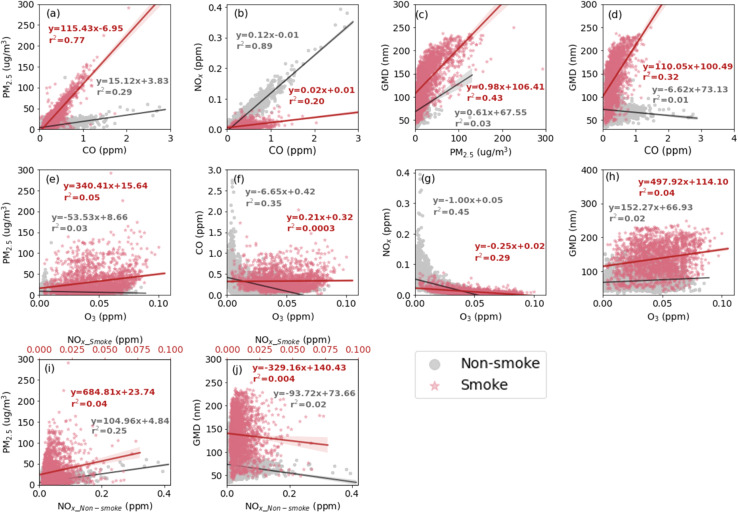
Correlation between PM_2.5_, CO, NO_*X*_, and O_3_ concentrations and GMD with linear regression lines during the smoke-influenced days (red dots) and the non-smoke-influenced days within the whole period (gray dots). The shadow around the linear regression line shows a 95% confidence interval. The linear regression information is annotated in the plot.

During smoke-influenced days, PM_2.5_ and CO concentrations had a good correlation (Fig. 5a) with a high *r*^2^ value (0.77, see Table 2), which is consistent with results from Jaffe *et al.*^74^ for data collected in Sparks, NV, which is located approximately 5 km to the northeast of the downtown Reno site. On smoke-influenced days, the relationship between PM_2.5_ and CO is clearly different from the relationship observed on non-smoke-influenced days (baseline + other) (Table 2). This indicates that the smoke-influenced days were influenced by a different air pollution source, which is yet another confirmation for the selection of smoke-influenced days.

**Table 2:** Slope, intercept, coefficient of determination (*r*^2^), slope uncertainty (slp_unc), and intercept uncertainty (int_unc) information of the linear regression lines between different air pollutant concentrations during smoke-influenced and non-smoke-influenced days (baseline + other). CO, NO_*X*_, and O_3_ units = ppm; PM_2.5_ unit = μg m^−3^; GMD unit = nm

Axis		Smoke-influenced days					Non-smoke-influenced days				
*y*	*x*	Slope	Intercept	*r* ^2^	slp_unc	int_unc	Slope	Intercept	*r* ^2^	slp_unc	int_unc
PM_2.5_	CO	115.43	−6.95	0.77	1.44	13.93	15.12	3.83	0.29	0.28	4.68
NO_*X*_	CO	0.02	0.01	0.20	0.00	0.01	0.12	−0.01	0.89	0.00	0.01
GMD	PM_2.5_	0.98	106.41	0.43	0.03	32.61	0.61	67.55	0.03	0.04	18.19
GMD	CO	110.05	100.49	0.32	3.68	35.73	−6.62	73.13	0.01	1.10	18.46
PM_2.5_	O_3_	340.41	15.64	0.05	35.56	28.49	−53.53	8.66	0.03	3.70	5.49
CO	O_3_	0.21	0.32	0.00	0.28	0.22	−6.65	0.42	0.35	0.11	0.16
NO_*X*_	O_3_	−0.25	0.02	0.29	0.01	0.01	−1.00	0.05	0.45	0.01	0.01
GMD	O_3_	497.92	114.10	0.04	52.81	42.31	152.27	66.93	0.02	12.34	18.31
PM_2.5_	NO_*X*_	684.81	23.74	0.04	78.18	28.60	104.96	4.84	0.25	2.20	4.84
GMD	NO_*X*_	−329.16	140.43	0.00	118.09	43.20	−93.72	73.66	0.02	8.33	18.34

In contrast, NO_*X*_ and CO showed good correlation on the non-smoke-influenced days (Fig. 5b) with a high *r*^2^ value (0.89). This is likely because urban emissions, such as those from traffic, dominated the relationship between these two pollutants on non-smoke-influenced days. In fact, the ratio of NO_*X*_ and CO (0.12 ppm ppm^−1^) is close to that of summer emissions from light-duty vehicles (0.134 ppm ppm^−1^) measured in the Fort McHenry Tunnel.^75^ The other pollutant pairs did not show any clear correlations during smoke-influenced days or during baseline and other days (Fig. 5e–j).

GMD during smoke-influenced days increased with PM_2.5_ concentrations (Fig. 5c). The non-smoke-influenced days showed a similar trend, although with a weaker dependence of GMD on PM_2.5_ concentration. A similar relationship between CO and GMD was observed during smoke-influenced days, with a much weaker relationship observed on non-smoke-influenced days.

**Fig. 6:**
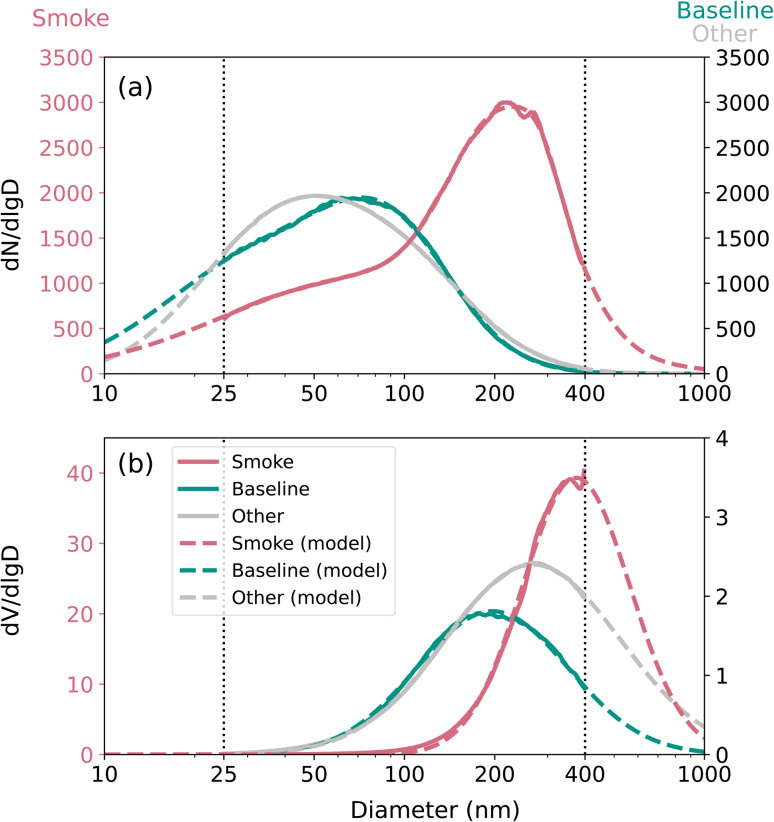
Particle (a) number and (b) volume distributions during smoke-influenced days, baseline days, and the other days. Red lines represent smoke-influenced days, green lines represent baseline days, gray lines represent the other days, solid lines represent the particle size distribution from ambient data, and dashed lines represent the modeled particle size distribution. The *y*-axis on the left is for smoke-influenced days, and the *y*-axis on the right is for baseline days and other days.

Fig. 6 shows the particle number distribution (Fig. 6a) and the particle volume distribution (Fig. 6b) during smoke-influenced days, baseline days, and other days. The figure also includes fitted multimode log-normal distributions and the fit parameters are provided in Table 3. During smoke-influenced days, the main mode of the number PSD (212 nm) was significantly greater than that during the baseline days and the rest of the campaign (74 nm and 48 nm, respectively). The volume PSD (339 nm) was also greater than during the baseline days (173 nm) and the other days (226 nm), which is in line with the observed differences in GMD (Fig. 4). Fig. 6 confirms that traffic and wildfires have different number and volume distributions, which is contrary to the results of Sandradewi *et al.*^76^

**Table 3:** Parameters of fitted normalized number (d*N*/d lg *D*) and volume (d*V*/d lg *D*) PSD during the smoke-influenced days, the baseline days, and the other days. GMD = geometric mean diameter; *σ*_g_ = geometric standard deviation; *N*_tot_ = total number concentration per mode, cm^−3^

		Mode	GMD	*σ*_g_	*N*_tot_
Number	Baseline	1	36.5	2.184	1161.3
		2	91.5	1.621	602.5
	Other	1	36.5	1.822	990.3
		2	94.1	1.791	734.9
	Smoke	1	71.1	2.703	1149.6
		2	228.5	1.534	1147.1
Volume	Baseline	1	196.5	1.784	1.14
	Other	1	267.9	1.955	1.75
	Smoke	1	373.6	1.513	17.72

The particle number distribution during smoke-influenced days in this study has the mode at diameters larger than the modeled young-plume distribution (59 to 94 nm) but comparable to the ∼1 to 2 days aged distributions (230 nm) in the study from Sakamoto *et al.*^77^ Our number PSD is also comparable with the number distribution mode at approximately 200 to 250 nm in the Williams Flats Fire for smoke with ages between 1.2 and 3.1 hours.^78^ However, the mean number PSD measured in our study is larger than the diameter of 140 nm observed during smoke-influenced days in Spain.^25^ This discrepancy could probably be due to variations in the distances between the wildfires and the measurement site. The volume PSD observed in this study during smoke-influenced days is smaller than the long-range transported BB aerosols measured in Europe with a volume distribution mode between 420 and 500 nm.^79^ However, the measurement range of our study did not extend that far to exclude the presence of a larger mode. Our observations are slightly larger than the mode at diameters between 220 and 300 nm reported for fire seasons in the Amazon Basin.^80^

The larger PSD during smoke-influenced days could enhance the Fine-mode Aerosol Optical Depth (FAOD), which at least in part could be a reason for the FAOD trend observed in the western United States, as demonstrated by Luo *et al.*^81^ The higher concentrations and larger sizes of wildfire-emitted aerosols could also potentially contribute to the formation of coarse mode aerosols through processes such as coagulation and condensation, thus influencing the Coarse-mode Aerosol Optical Depth (CAOD). However, since our measurements were limited to aerosols with diameters smaller than 400 nm, such contributions cannot be confirmed.

### 3.2 Factor analysis

**Fig. 7:**
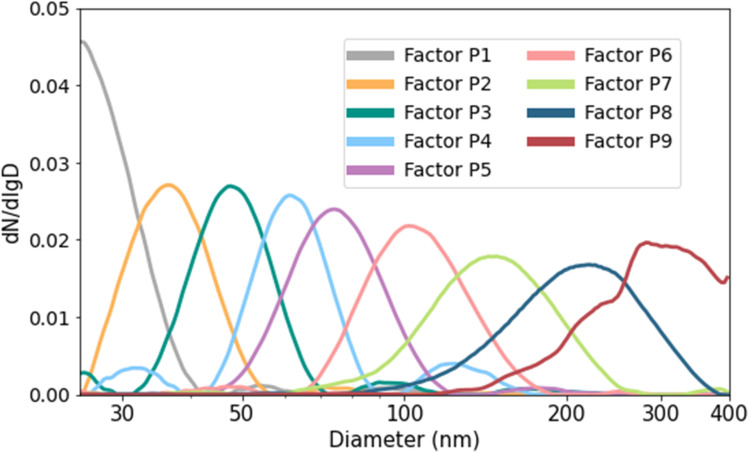
The normalized PSD of each factor in a nine-factor analysis during the whole study period.

To investigate whether specific sources, including wildfires, have a characteristic PSD, the collected PSD data was analyzed using a home-written Python PMF program. The PMF was run for 5 to 13 factors, and 9 factors were chosen for the analysis presented in this paper because a higher number of factors provides only a marginal improvement in explaining the observed variability (Fig. S1†). Fig. 7 shows the PSD factors. These factors were then analyzed to assess whether they correspond to different PM sources, such as wildfires and traffic. It should be noted that the PMF results were found to be robust to measurement uncertainties, which was checked using 100 Monte Carlo simulations, where the input PSD were perturbed by 10% random errors (Fig. S2†).

In addition to the PSD analysis, the EPA PMF was used to analyze daily pollutant concentrations (including PM_2.5_, CO, O_3_, NO, NO_2_, K, EC, and OC) collected from December 2015 to May 2022. The PMF factors were then fitted to hourly chemical data (including PM_2.5_, CO, O_3_, NO, NO_2_) using non-negative least squares (NNLS) to obtain hourly contributions of each of the PMF factors. Four factors were selected based on the results of error estimation analysis: no swap occurred in DISP, all factors had 100% mapping in BS, and a change in the goodness-of-fit parameter *Q* was less than 0.5% with no swap in BS-DISP.

**Fig. 8:**
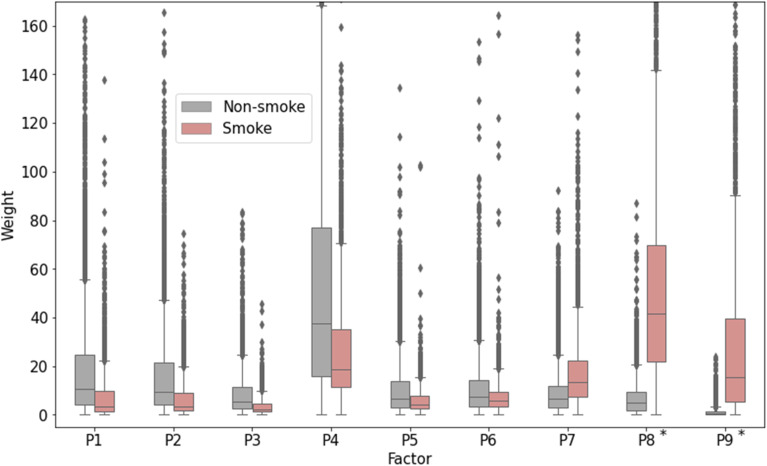
The boxplot of the contribution (weight) for each factor during smoke-influenced days (red) and non-smoke-influenced (baseline + other) days (gray). Wildfire-related factors are marked by an asterisk (*).

Fig. 8 shows a comparison of PSD factor contributions during smoke-influenced days and all the other days 8. The letters “P” and “E” in front of the factor numbers stand for “PSD” and “EPA,” respectively, to distinguish factors from the Python PMF program using PSD data and the EPA PMF program using air pollutant concentrations. Contributions of Factor P8 and Factor P9 during the smoke-influenced days are significantly higher (*p* < 0.001) than during the other days, and therefore are likely to represent fire-emitted PSD. These factors have the largest modes and GMD, which agrees with the observation that the smoke-influenced days tended to have the mode and GMD at larger particle sizes than the baseline or other days (Fig. 4 and 6). The other factors likely represent PSD from traffic and other sources. Factor P7 had a slightly higher contribution during the smoke-influenced days than the other days, but it was excluded as a wildfire-related factor because it was considered representing a mixed source – such as a mixture of wildfire emissions, traffic emissions, and domestic wood-burning emissions – or that several sources could have a similar PSD.

**Fig. 9:**
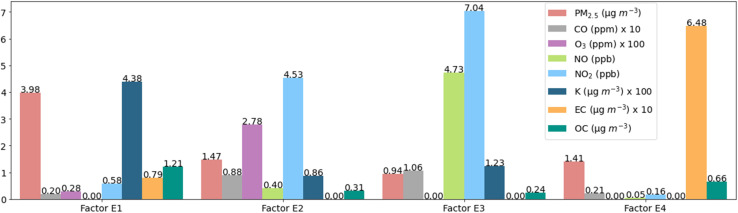
Species profile of the EPA PMF for all four factors. The approximated values of the profile are displayed on the top. O_3_, K, and EC were multiplied by 10 or 100 to make them easier to see.

Based on the species profile (Fig. 9; a detailed profile plot is provided in Fig. S3,† the uncertainty estimates are shown in Fig. S4†) and the contribution plots (Fig. S5†) from the EPA PMF, Factors 1 to 4 represent different emission sources. Factor E1, which is the EPA PMF wildfire-related factor, had the highest PM_2.5_ concentration along with the highest potassium and OC concentration. Potassium is often used as a tracer for wildfire emissions.^82^ The profile of Factor E2 is associated with ozone and had significant contributions during summer and low contributions during winter, indicating that this profile represents secondary sources. Factor E3 contributions showed a clear increase and decrease in winter and summer, respectively, following the variation of the vertical atmospheric mixing. The profile of Factor E3 displayed the highest values of NO_*X*_ (NO + NO_2_) and CO. This suggests that Factor E3 is a traffic-related factor. Factor E4 had a similar contribution plot as Factor E1 but with higher contributions during winter and lower contributions during smoke-influenced months in 2017 and 2018. It had the highest EC, some OC and PM_2.5_, and a small amount of CO and NO_2_. This suggests that Factor E4 is a mixture of domestic wood burning and wildfire emissions. According to the results from the EPA PMF, during fire season (July, August, and September), wildfires (Factor E1) contributed 56% to 65% to PM_2.5_ concentrations, 18% to 26% to CO concentrations (Fig. S6†). This also demonstrates the significant contribution of wildfires to PM_2.5_ concentrations during the fire season.

**Fig. 10:**
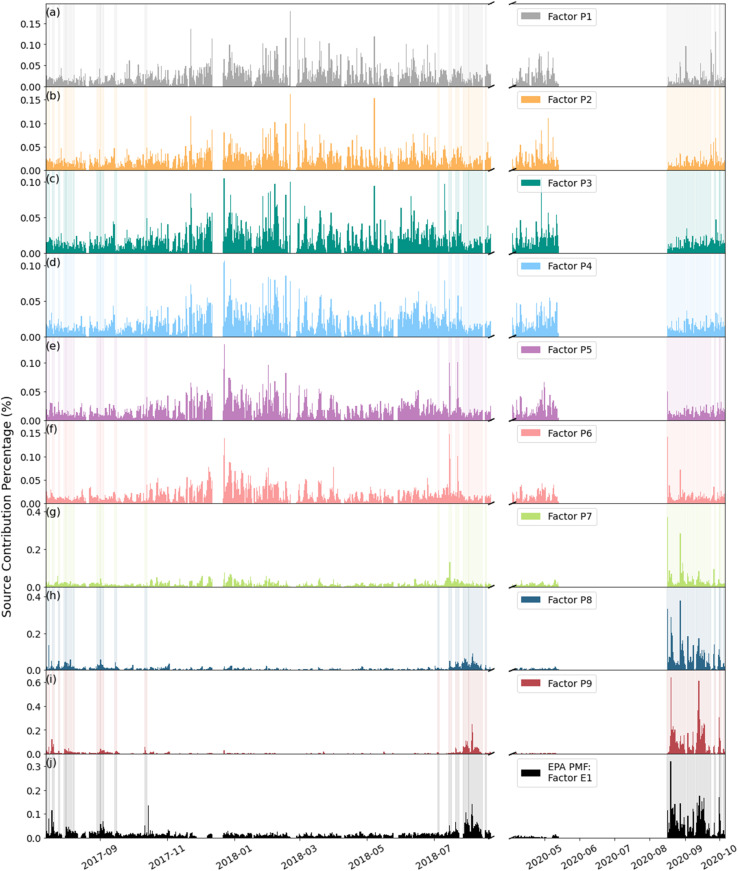
Hourly contribution plots of (a)–(i) factors from PSD PMF and (j) the wildfire-related factor from EPA PMF. The shadowed area represents the smoke-influenced days.

The contribution of the wildfire-related factor (Factor E1) from the EPA PMF was compared with the nine PSD factors to confirm the relationship between PSD and wildfires (Fig. 10). The temporal variability of the two wildfire-related PSD factors (Factors P9 and P8) had strong correlations with the temporal variability of the EPA PMF wildfire-related factor (Fig. 10j), with correlation coefficients of 0.84 and 0.72 respectively. This further confirms that Factor P9 and Factor P8 are very likely wildfire related. The differences between Factor P8 and Factor P9 could be caused by different distances between fires and the measurement location: a larger distance from fire can lead to larger particles due to the growth and formation of particles during the transport.^83,84^ In this study, isolation and quantification of distance-specific effects was challenging due to the frequent concurrent influence of multiple wildfires at varying distances on the measurement site. This will be the subject of a follow-up study.

Factor P7 in PSD PMF had similar contributions in August 2020 as the EPA PMF wildfire-related factor, but it had higher contributions during winter compared to the EPA PMF wildfire-related factor, which suggested that it contained other emission sources in winter, such as traffic and domestic wood burning. In addition, the correlation coefficient between Factor P7 and the EPA PMF wildfire-related factor was rather low (0.33). Therefore, Factor P7 likely represents a mixture of different sources. All non-fire-related PSD PMF factors did not match the EPA PMF wildfire-related factor (the correlation coefficient ranged from −0.14 to −0.0048). It should be noted that the Python PMF applied to the EPA data produces fire-related factors that correlate well with those from the EPA PMF (Fig. S7†). This further confirms that the two factors with the largest GMD are associated with wildfire emissions.

**Fig. 11:**
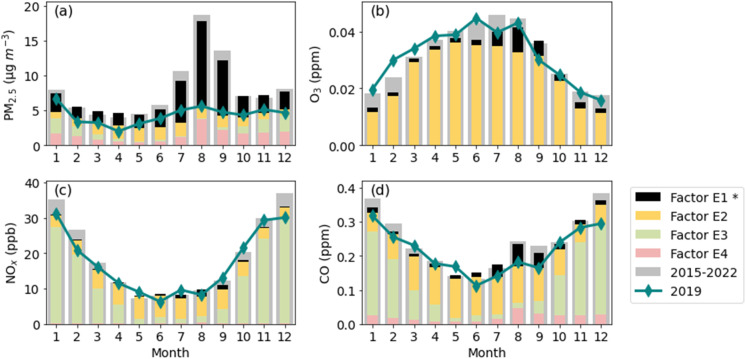
Monthly averaged daily pollutant ((a) PM_2.5_, (b) O_3_, (c) NO_*X*_, and (d) CO) concentrations during 2015 to 2022 with EPA PMF contributions of each factor and 2019 in Reno, NV. The wildfire-related factor is indicated by an asterisk (*).

The impact of wildfires on air quality in Reno, NV, is notable when observing the monthly averaged data of PM_2.5_ and other air pollutant concentrations averaged over 6.5 years (December 2015 to May 2022), as presented in Fig. 11. The contributions of each factor from EPA PMF are also shown in the plot. The differences between the sum of factor contributions and the ambient concentrations are due to the residual matrix (*E*). For comparison, the pollutant concentrations measured in 2019, a relatively smoke-free year, are also shown in Fig. 11 to indicate monthly variations with little to no wildfire impact.

During fire months (mainly July, August, and September in Reno, NV), especially during August and September, the mean PM_2.5_ concentration showed a clear increase over the 2019 concentration (by 113%, 234%, and 186% in July, August, and September, respectively), with the difference closely matching the contribution of the wildfire factor and the average sum of three non-wildfire factors (Factor E2, E3, and E4) being comparable to the 2019 values. Winter PM_2.5_ monthly mean concentrations were high, but still lower than concentrations during the fire months. The seasonal profile of PM_2.5_ concentrations in the winter followed the atmospheric mixing-driven influence of local sources.^85^ On a yearly basis, wildfires contributed 35–47% of PM_2.5_ concentrations in Reno, NV from 2016 to 2021 (the data for 2015 and 2022 covers less than a full year).

The highest concentrations of O_3_ were in summer (June, July, and August) and the lowest concentrations were in winter. The seasonal variations of O_3_ follow the photochemical activity,^86–88^ which is strongest in summer and weakest in winter. Wildfire emissions had a smaller contribution to O_3_ than to PM_2.5_ concentrations, as indicated by Factor E1, with increases ranging from 11% to 20% from July to September. This finding aligns with the observations reported by McClure & Jaffe.^89^ Wildfire emissions can contribute to photochemical reactions,^90^ which could contribute to O_3_ production. The yearly contribution of wildfire emissions to O_3_ concentration is estimated to be 6% to 13% during 2016–2021.

The increases in CO concentrations attributable to wildfire contributions were also relatively modest, ranging from 18% to 26% from July to September and 5% to 12% on an annual basis from 2016 to 2021. In contrast, no significant change was observed in the NO_*X*_ concentration trend. In winter (December, January, and February), especially in December and January, NO_*X*_ and CO concentrations were highest, reaching 36.89 ppb and 0.38 ppm in December, respectively. In general, NO_*X*_ and CO seasonal profiles could be explained by seasonal variation in vertical atmospheric mixing. During winter months, atmospheric mixing is weakest,^85^ leading to the accumulation of pollution from local combustion sources, such as traffic.

## 4. Conclusion

In this study, ambient measurements in Reno, NV, from July 2017 to October 2020 were used to identify differences in PSD and air pollutant concentrations between smoke-influenced days and the non-smoke-influenced days. A PMF method was applied to identify the main source contributions, as well as the PSD and pollutant profiles corresponding to these sources. Wind back-trajectories and satellite images were used to identify the smoke-influenced days.

A comparison between smoke-influenced and non-smoke-influenced days shows that wildfires have a significant influence on PSD and air pollutant concentrations. In this study, the smoke-influenced days were characterized by substantially and statistically significantly larger GMD and higher *V*_tot_ and PM_2.5_ concentrations than the non-smoke-influenced days. The average number GMD during the smoke-influenced days (139 nm) was approximately two times larger than that during the non-smoke-influenced days (72 nm). The smoke-influenced days showed an average PM_2.5_ concentration (31 μg m^−3^) approximately four times higher than that of non-smoke-influenced days (7 μg m^−3^).

The PMF techniques successfully separated wildfire emissions from other sources using both PSD data and the air pollutant concentrations. During the fire months (July, August, and September), between 52% to 58% of PM_2.5_ concentrations, 14% to 23% of CO concentrations, and 10% to 19% of O_3_ concentrations were attributed to wildfire emissions according to the PMF analysis. On an annual basis, wildfire emissions contributed 35% to 47% of PM_2.5_ concentrations, 5% to 12% of CO concentrations, and 6% to 13% of O_3_ concentrations.

## Data availability

Part of the data supporting this article (information on fires) has been included as part of the ESI.† Particle size distribution data used in this article are available at Zenodo at https://doi.org/10.5281/zenodo.14740744.

## Author contributions

Siying Lu: writing – original draft, writing – review & editing, conceptualization, formal analysis, software, visualization. Chiranjivi Bhattarai: data curation, investigation. Vera Samburova: writing – review & editing, funding acquisition. Andrey Khlystov: writing – original draft, writing – review & editing, conceptualization, software, supervision, funding acquisition.

## Conflicts of interest

There are no conflicts to declare.

## Supplementary Material

EA-005-D5EA00007F-s001
