# Supplementary material for: Particle size distributions of wildfire aerosols in the western USA
Source: Environ Sci Atmos. 2025 Mar 13;5(4):502–16. doi: 10.1039/d5ea00007f (PMC11917463; doi:10.1039/d5ea00007f)
Supplement: EA-005-D5EA00007F-s001 [file EA-005-D5EA00007F-s001.pdf]

Supplementary Information (SI) for Environmental Science: Atmospheres.
This journal is © The Royal Society of Chemistry 2025

Supplementary material

Particle Size Distributions of Wildfire Aerosols in the Western USA

Siying Lu[1], Chiranjivi Bhattarai[1], Vera Samburova[1,2], Andrey Khlystov[1,*]

[1]Division of Atmospheric Sciences, Desert Research Institute, Reno, Nevada, USA;

[2]Department of Physics, University of Nevada, Reno, Nevada, USA

*Corresponding author. Email address: andrey.khlystov@dri.edu (A. Khlystov)

Table S1 Information of wildfires with potential to impact Reno, NV during 2017, 2018, and 2020.

Fire name	Start day	End day	Burned area (acres)	County	Location	Lat/Lon	Ecosystem	Corresponding Vegetation
Salmon August Complex Fire	6/25/2017	12/8/2017	65,888	Siskiyou, CA	14 miles west of Etna, California in the Marble Mountain Wilderness	41.841, -123.474	Klamath Subalpine; Marble/Salmon Mountains-Trinity Alps	Shasta red fir, mountain hemlock, western white pine, subalpine meadows, mixes of shrubs, herbs, and grasses, foxtail pine, Pacific silver fir; Douglas-fir, white fir, and red fir.
Whittier Fire	7/8/2017	10/5/2017	18,430	Santa Barbara, CA	In the Santa Ynez Mountains, Los Padres National Forest, south of Lake Cachuma, along Highway 154	34.567, -119.953	Solomon-Purisima-Santa Ynez Hills; Western Transverse Range Lower Montane Shrub and Woodland	Coastal sage scrub, chaparral, oak woodland, grasslands; coast live oak, canyon oak, Coulter pine, California bay, bigcone Douglas-fir, chamise, ceanothus, manzanita, scrub oaks, cherry, and birchleaf mountain-mahogany
Garza Fire	7/9/2017	1/9/2018	48,889	King, CA	Tar Canyon Road, near Avenal	35.933, -120.200	Pleasant Valley/Kettleman Plain	Cropland, redstem filaree, soft chess, red brome, foxtail barley, and burclover.
Long Valley Fire	7/11/2017	7/21/2017	83,733	Lassen, CA; Washoe, NV	Off Hwy 395 and Cowboy Joe Rd, 3 miles north of Doyle, CA and blew east towards Pyramid Lake	40.070, -120.140	Lahontan and Tonopah Playas; Sierra Nevada-Influenced Semiarid Hills and Basins; Sierra Nevada-Influenced Ranges	Black greasewood, four-winged saltbush, and salt shrub community; antelope bitterbrush, desert peach, semiarid shrub community, and riparian cottonwood forest; pinyon–juniper woodland, ponderosa, lodgepole, Jeffrey, and western white pine
Chetco Bar Fire	7/12/2017	11/2/2017	191,125	Curry, OR	In the Kalmiopsis Wilderness near the Chetco River	42.297, -123.954	Coastal Siskiyous; Serpentine Siskiyous; Southern Oregon Coastal Mountains	Mixed conifer forest, Douglas-fir, ponderosa pine, Oregon white oak, California black oak, madrone, serviceberry, snowberry, Oregon grape, California fescue, and poison oak; Jeffrey pine, tanoak, incense cedar, chaparral composed of manzanita, ceanothus, Idaho fescue, and Lemmon needlegrass; Hemlock canopy, salal, sword fern, vine maple, rhododendron shrub, California bay-laurel, western redcedar, bigleaf maple, grand fir, red alder, salmonberry and oxalis

Table S1 (Continued)

Fire name	Start day	End day	Burned area (acres)	County	Location	Lat/Lon	Ecosystem	Corresponding Vegetation
Cold Springs Fire	7/14/2017	7/15/2017	1,523	Washoe, NV	Southwest of Clod Springs, NV		Sierra Nevada-Influenced Semiarid Hills and Basins	Wyoming big sagebrush, antelope bitterbrush, desert peach, low sagebrush, Lahontan sagebrush, Douglas rabbitbrush, Nevada ephedra, bottlebrush squirreltail, desert needlegrass, Thurber needlegrass, Indian ricegrass, cheatgrass, bluebunch wheatgrass, Sandberg bluegrass, juniper, Riparian cottonwood woodland, buffaloberry.
Detwiler Fire	7/16/2017	8/24/2017	81,826	Mariposa, CA	Detwiler Rd and Hunters Valley Rd, 2 miles east of Lake McClure	37.617, -120.213	Northern Sierran Foothills; Central Sierra Lower Montane Forests	Needlegrass, annual grasslands, chamise, manzanita, interior live oak, ceanothus, blue oak, and foothill pine; Ponderosa pine, gray pine, mixed conifer forest, Montane hardwood forests, canyon live oak, black oak, shrubby tanoak, mixed chaparral, manzanita, ceanothus, and other shrubs
Whitewater Fire	7/23/2017	11/21/2017	14,451	Clackamas, OR; Marion, OR	In and near the Mount Jefferson Wilderness, approximately 13 miles east of Detroit, OR	44.708, -121.856	Western Cascades Lowlands and Valleys; Western Cascades Montane Highlands	Douglas-fir, western hemlock, western redcedar, bigleaf maple, red alder, vine maple, salal, rhododendron, Oregon grape, huckleberry, thimbleberry, swordfern, oxalis, hazel, and blackberry; Pacific silver fir, mountain hemlock, noble fir, bigleaf maple, and Pacific yew
Preacher Fire	7/24/2017	7/29/2017	5,333	Douglas, NV	East of Highway 395 about 7 miles from the town of Gardnerville, NV		Sierra Nevada-Influenced Semiarid Hills and Basins	Wyoming big sagebrush, antelope bitterbrush, desert peach, low sagebrush, Lahontan sagebrush, Douglas rabbitbrush, Nevada ephedra, bottlebrush squirreltail, desert needlegrass, Thurber needlegrass, Indian ricegrass, cheatgrass, bluebunch wheatgrass, Sandberg bluegrass, scattered juniper, Riparian cottonwood woodland, buffaloberry.
Modoc July Complex Fire	7/24/2017	1/9/2018	83,120	Modoc, CA	Throughout Modoc County		Klamath Juniper Woodland/Devils Garden; Fremont Pine/Fir Forest; Adin/Horsehead Mountains Forest and Woodland	Western juniper, low sagebrush, mountain big sagebrush, bitterbrush, bunchgrasses, woolly mule-ears, Klamath plum, and birchleaf mountain-mahogany; Closed-canopy forests, Ponderosa pine, white fir, sugar pine, lodgepole pine, and incense cedar; Jeffrey pine, and other deciduous shrubs.

Table S1 (Continued)

Fire name	Start day	End day	Burned area (acres)	County	Location	Lat/Lon	Ecosystem	Corresponding Vegetation
Eclipse Complex Fire	7/25/2017	1/9/2018	100,000+	Siskiyou, CA	5 miles W of Happy Camp Happy Camp, CA, in the Siskiyou Mountains	41.841, -123.474	Serpentine Siskiyous; Western Klamath Low Elevation Forests; Western Klamath Montane Forests	Jeffrey pine, endemic oak, ceanothus species, forbs, Douglas-fir, and western white pine; Port Orford cedar, tanoak, canyon live oak, red alder, white alder, mixed oak
High Cascades Complex Fire	7/26/2017	11/30/2017	72,309	Douglas, OR; Jackson, OR	Crater Lake National Park, Rogue River-Siskiyou National Forest, Umpqua National Forest, and Fremont–Winema National Forest	42.935, -122.407	High Southern Cascades Montane Forest; Cascades Subalpine/Alpine; Southern Cascades	Mountain hemlock, lodgepole pine, Pacific silver fir, grand fir, white fir, Shasta red fir, woodrush, prince’s pine, lupine, sidebells shinleaf, white bark pine, Shasta buckwheat, Newberry knotweed, and Brewer’s sedge; alpine meadows, barren, Herbaceous and shrubby subalpine meadow, subalpine fir, Holm’s sedge, black alpine sedge, tufted hairgrass, and alpine aster; Douglas-fir, ponderosa pine, incense cedar, sugar pine, snowberry, twinflower, Oregon grape, serviceberry, golden chinkapin, and oceanspray.
Orleans Complex Fire	7/26/2017	1/9/2018	27,276	Siskiyou, CA	About 18 miles northwest of Orleans, California in Siskiyou County	41.59, -123.501	Western Klamath Low Elevation Forests	Douglas-fir, Port Orford cedar, tanoak, canyon live oak, red alder, white alder, mixed oak
Minerva Fire	7/29/2017	8/17/2017	4,310	Plumas, CA	Forest Road 24N26, 3 miles southwest of Quincy	39.903, -120.976	Northern Sierra Upper Montane Forests; Northern Sierra Mid-Montane Forests	Red fir, white fir, Jeffrey pine, sugar pine, incense cedar, lodgepole pine, quaking aspen, and montane chaparral; mixed conifer forest, Douglas-fir, ponderosa pine, black oak, tanoaks, and canyon live oak
Largo Fire	7/30/2017	1/9/2018	236	El Dorado, CA	South Shingle Rd at Michigan Bar Rd, northeast of Rancho Murieta	38.538, -121.039	Northern Sierran Foothills	Needlegrass, annual grasslands, chamise, manzanita, interior live oak, ceanothus, blue oak, and foothill pine.
Devils Lake Fire	7/31/2017	8/10/2017	1,702	Klamath, OR	Primarily on the Bly Ranger District, Fremont-Winema National Forest, but is moving south into more heavily-timbered terrain protected by Oregon Department of Forestry.		Klamath Juniper Woodland	Ponderosa pine, antelope bitterbrush, bunchgreasses, low sagebrush, Wyoming big sagebrush, Idaho fescue, bluebunch wheatgrass, Sandberg bluegrass, Western juniper, mountain-mahogany.

Table S1 (Continued)

Fire name	Start day	End day	Burned area (acres)	County	Location	Lat/Lon	Ecosystem	Corresponding Vegetation
Summit Complex Fire	7/31/2017	1/9/2018	5,247	Stanislaus, CA	The Summit Complex consists of 3 fires burning on the Summit Ranger District of the Stanislaus National Forest	38.329, -119.782	Central Sierra Mid-Montane Forests	Mesic mixed conifer forest, Douglas-fir, ponderosa pine, canyon, live, and black oaks
Empire Fire	8/1/2017	11/27/2017	8,094	Mariposa, CA	East of Badger Pass Ski Area and next to Bridalveil Creek Campground in Yosemite National Park, CA	37.644, -119.618	Southern Sierra Upper Montane Forests	Mixed conifer, white fir, red fir forests, Jeffrey pine, lodgepole pine, montane chaparral
Cinder Butte Fire	8/2/2017	8/17/2017	52,531	Lake, OR; Harney, OR	In the area east of Glass Buttes in northern Lake County and then spread into northwestern Harney County	43.578, -119.94	Semiarid Uplands; High Lava Plains	Mountain big sagebrush, low sagebrush, mountain-mahogany, Idaho fescue, Sandberg bluegrass, Nevada bluegrass, bluebunch wheatgrass, bottlebrush squirreltail, Thurber needlegrass, western juniper woodland, aspen, chokecherry, willows; Wyoming big sagebrush, silver sagebrush, creeping wildrye, mat muhley
Indian Fire	8/2/2017	1/9/2018	2,295	Tulare, CA	North of Jordan Hot Springs	36.257, -118.296	Southern Sierra Mid-Montane Forests	Ponderosa pine forests, mixed conifer forest, Jeffrey pine, giant sequoia
Parker 2 Fire	8/3/2017	8/28/2017	7,697	Modoc, CA	Southwest of Cedarville, in the Modoc National Forest	41.466, -120.321	Modoc Lava Flows and Buttes	Western juniper, big sagebrush, and native perennial grassland
Saw Fire	8/3/2017	1/9/2018	85	Humboldt, CA	Off Alderpoint Rd & Lower Sawmill Rd, east of Garberville	40.118, -123.761	Outer North Coast Ranges	Douglas-fir, tanoak, Oregon white oak, needlegrass grasslands, redwood
Calgary Fire	8/5/2017	8/8/2017	50	Kern, CA	Old State Rd & Dollar Rd, Wofford Heights	35.706, -118.473	Tehachapi Foothills	Blue oak, needlegrass, annual grasslands, chamise, ceanothus, mixed oaks, and foothill pine
Poslin Fire	8/6/2017	8/9/2017	859	Lassen, CA	Scott Rd, 3 miles northeast of Scotts, 9 miles south of Doyle	39.888, -120.066	Northeastern Sierra Mixed Conifer-Pine Forests	Jeffrey, ponderosa, sugar pines, incense cedar, California white fir, sagebrush, antelope bitterbrush, snowbrush and manzanita
Falcon Complex Fire	8/8/2017	9/15/2017	2,935	Douglas, OR	Umpqua National Forest		Western Cascades Lowlands and Valleys	Douglas-fir, western hemlock, western redcedar, bigleaf maple, red alder, vine maple, salal, rhododendron, Oregon grape, huckleberry, thimbleberry, swordfern, oxalis, hazel, and blackberry

Table S1 (Continued)

Fire name	Start day	End day	Burned area (acres)	County	Location	Lat/Lon	Ecosystem	Corresponding Vegetation
Nena Springs Fire	8/8/2017	8/29/2017	68,135	Jefferson, OR	14 miles north of Madras		John Day/Clarno Uplands; Deschutes River Valley; Deschutes/John Day Canyons; Umatilla Plateau	Bluebunch wheatgrass, Idaho fescue, basin wildrye, Wyoming big sagebrush, Thurber needlegrass, Western juniper woodland, ponderosa pine forest, white alder, mockorange, chokecherry, clematis, willows, black cottonwood, water birch; Antelope bitterbrush, basin big sagebrush, mountain big sagebrush, needleandthread, black hawthorn, juniper, Himalayan blackberry, reed canarygrass, and sweet clover; Sandberg bluegrass, cheatgrass, western clematis; stiff sagebrush, alien cheatgrass.
Staley	8/10/2017	10/31/2017	2,300	Lane, OR	In Willamette National Forest		Western Cascades Montane Highlands	Pacific silver fir, western hemlock, mountain hemlock, Douglas-fir, noble fir, bigleaf maple, red alder, Pacific yew, vine maple, rhododendron, Oregon grape, huckleberry, and thimbleberry
Jones Fire	8/10/2017	11/29/2017	10,114	Lane, OR	In Willamette National Forest, approximately 10 miles northeast of Lowell	44.003, -122.575		
Milli Fire	8/11/2017	9/24/2017	24,079	Deschutes, OR	Deschutes National Forest	44.257, -121.743	Ponderosa Pine/Bitterbrush Woodland	Ponderosa pine, antelope bitterbrush, greenleaf manzanita, snowberry, mountain alder, stream dogwood, willows, and sedges.
Umpqua North Complex Fire	8/11/2017	10/30/2017	43,158	Douglas, OR	Along Oregon Highway 138 east of Roseburg and in the northern part of the Umpqua National Forest		Western Cascades Lowlands and Valleys; Western Cascades Montane Highlands	Douglas-fir, western hemlock, western redcedar, bigleaf maple, red alder, vine maple, salal, rhododendron, Oregon grape, huckleberry, thimbleberry, swordfern, oxalis, hazel, and blackberry; Pacific silver fir, mountain hemlock, noble fir, bigleaf maple, Pacific yew.
Horse Prairie Fire	8/26/2017	9/29/2017	16,436	Douglas, OR	Southwest of Roseburg, 12 miles west of Riddle and 8 miles southeast of Camas Valley		Inland Siskiyous	Douglas-fir, ponderosa pine, Oregon white oak, California black oak, madrone, serviceberry, snowberry, Oregon grape, California fescue, and poison oak.

Table S1 (Continued)

Fire name	Start day	End day	Burned area (acres)	County	Location	Lat/Lon	Ecosystem	Corresponding Vegetation
Tungsten Fire	8/27/2017	9/14/2017	22,000	Churchill, NV	60 miles east of Fallon, NV	39.731, -117.725	Central Nevada Mid-Slope Woodland and Brushland	Singleleaf pinyon, Utah juniper, black sagebrush, Wyoming big sagebrush, green ephedra, rabbitbrush, desert bitterbrush, Thurber needlegrass, Sandberg bluegrass, bluebunch wheatgrass, Idaho fescue, cheatgrass, mountain big sagebrush, curlleaf mountain-mahogany, cottonwood, aspen, water birch, chokecherry, and coyote willow.
Mud Fire	8/29/2017	9/1/2017	6,042	Lassen, CA	Little Mud Flat, 18 miles northeast of Susanville	40.440, -120.222	High Lava Plains	Wyoming big sagebrush, Bluebunch wheatgrass, cheatgrass
Tohakum 2 Fire	8/29/2017	9/7/2017	94,221	Washoe, NV	Tohakum Peak northeast of Pyramid Lake		Lahontan Uplands; Lahontan Sagebrush Slopes	Utah juniper, Singleleaf pinyon, mountain big sagebrush, Wyoming big sagebrush, black sagebrush, low sagebrush, green ephedra, antelope bitterbrush, rabbitbrush, Sandberg bluegrass, Thurber needlegrass, and bluebunch wheatgrass, Indian ricegrass; Lahontan sagebrush, littleleaf horsebrush, desert needlegrass, bottlebrush squirreltail, cheatgrass
Slinkard Fire	8/29/2017	9/12/2017	8,925	Mono, CA	West of Hwy 395, north of Hwy 89, south of Topaz	38.655, -119.554	Sierra Nevada-Influenced Ranges; Northeastern Sierra Mixed Conifer-Pine Forests; Sierra Nevada-Influenced Semiarid Hills and Basins	Pinyon–juniper woodland, ponderosa, lodgepole, Jeffrey, and western white pine; sugar pines, incense cedar, California white fir, sagebrush, antelope bitterbrush, snowbrush and manzanita; desert peach, semiarid shrub, riparian cottonwood forest, grazing, agriculture, and invasive weeds
Pier Fire	8/29/2017	11/29/2017	36,556	Tulare, CA	Highway 190, east of Springville	36.153, -118.741	Southern Sierran Foothills; Southern Sierra Lower Montane Forest and Woodland; Southern Sierra Mid-Montane Forests; Southern Sierra Upper Montane Forests	Blue oak, needlegrass and annual grasslands, chamise, ceanothus, mixed oaks, and foothill pine; chaparral, ponderosa pine; mixed conifer forest, Jeffrey pine, giant sequoia grove; white fir, red fir forests, lodgepole pine, montane chaparral

Table S1 (Continued)

Fire name	Start day	End day	Burned area (acres)	County	Location	Lat/Lon	Ecosystem	Corresponding Vegetation
Helena-Fork Fire	8/30/2017	11/15/2017	21,846	Trinity, CA	Hwy 299 near town of Helena, Junction City	40.760, -123.100	Eastern Klamath Low Elevation Forests; Eastern Klamath Montane Forests; Marble/Salmon Mountains-Trinity Alps	Douglas-fir, ponderosa pine, canyon live oak, knobcone pine, chaparral of chamise, deer brush, and manzanita, streams, cottonwoods, white alder, and willows; White fir, incense cedar, sugar pine, mountain dogwood, Shasta fir, red fir, Black oak, conifers, huckleberry oak
R-4 Fire	8/30/2017	1/9/2018	18,618	Lassen, CA	23 miles east-southeast of Madeline	40.696, -119.935	High Lava Plains	Bluebunch wheatgrass, Wyoming big sagebrush, cheatgrass, sagebrush
Buck Fire	9/12/2017	11/20/2017	13,417	Trinity, CA	Black Rock Mountain, North Portion of Yolla BollyWilderness	40.227, -123.036	High North Coast Ranges	Mixed conifer, Douglas-fir forests, tanoak, White fir forest, red fir
Cascade Fire	10/8/2017	10/19/2017	9,989	Yuba, CA	Cascade Way and Marysville Rd, north of Collins Lake	39.322, -121.402	Northern Sierran Foothills	Needlegrass, annual grasslands, chamise, manzanita, interior live oak, ceanothus, blue oak, and foothill pine
Redwood Valley Fire	10/8/2017	10/28/2017	36,523	Mendocino, CA	North of Hwy 20, west of Mendocino National Forest, south of Black Bart	39.249, -123.166	Coastal Franciscan Redwood Forest; Outer North Coast Ranges; Napa-Sonoma-Russian River Valleys	Multi-story canopy of redwood, Douglas-fir, tanoak, bigleaf maple, evergreen shrubs, various grasses, coast live oaks, and grassland savannas; Oregon white oak, redwood; Grape vineyards, orchards, specialty crops
Tubbs Fire	10/8/2017	10/31/2017	36,807	Napa, CA; Sonoma, CA	Off of Hwy 128 and Bennett Ln, Calistoga	38.609, -122.629	Napa-Sonoma-Lake Volcanic Highlands; Napa-Sonoma-Russian River Valleys; Mayacmas Mountains	Mixed chaparral, mixed hardwoods (including coast live oak, madrone, and California bay), Douglas-fir or pines, cypress; Grape vineyards, orchards, specialty crops; Annual grasslands, montane hardwoods, blue oak woodland, Sargent or McNab cypress
Atlas Fire	10/8/2017	10/28/2017	51,057	Napa, CA	North of the city of Napa, near Napa Soda Springs	38.392, -122.243	Napa-Sonoma-Lake Volcanic Highlands; Napa-Sonoma-Russian River Valleys; North Coast Range Eastern Slopes; Foothill Ridges and Valleys	Mixed chaparral, mixed hardwoods (including coast live oak, madrone, and California bay), Douglas-fir or pines, cypress; Grape vineyards, orchards, specialty crops; leather oak, chamise, mixed conifer, McNab or Sargent cypress, foothill and knobcone pine, blue oak woodland; purple needlegrass

Table S1 (Continued)

Fire name	Start day	End day	Burned area (acres)	County	Location	Lat/Lon	Ecosystem	Corresponding Vegetation
LaPorte Fire	10/9/2017	10/19/2017	6,151	Butte, CA	La Porte Rd and Oro Bangor Hwy, Bangor	39.394, -121.406	Northern Terraces	Annual grass, forbs, blue oak woodlands
Pocket Fire	10/9/2017	10/31/2017	17,357	Sonoma, CA	Off of Pocket Ranch Rd and Ridge Ranch Rd, Geyserville	38.765, -122.909	Mayacmas Mountains	Annual grasslands, montane hardwoods, mixed chaparral, blue oak woodland, Sargent or McNab cypress, Douglas-fir
Oakmont Fire	10/14/2017	4/5/2018	100	Sonoma, CA	East side of Highway 12 near the Oakmont community	38.461, -122.581	Napa-Sonoma-Russian River Valleys	Grape vineyards, orchards, specialty crops.
Lions Fire	6/11/2018	10/1/2018	13,347	Madera, CA	Near the Lion Point area in the Ansel Adams Wilderness	37.571, -119.118	Southern Sierra Mid-Montane Forests; Southern Sierra Upper Montane Forests; Southern Sierra Lower Montane Forest and Woodland; Sierran Alpine; Southern Sierra Subalpine Forests	Ponderosa pine forests, mixed conifer forest, Jeffrey pine, giant sequoia, white fir, red fir forests, lodgepole pine, montane chaparral, oak, chaparral, Cushion plants, tufted grasses, alpine willows, sedges, various shrubs, herbaceous plants, mountain hemlock, whitebark pine, western white pine, foxtail pine, and limber pine
Ferguson Fire	7/13/2018	8/19/2018	96,901	Mariposa, CA	Highway 140 and Hite Cove, near El Portal	37.655, -119.886	Southern Sierra Mid-Montane Forests; Southern Sierra Upper Montane Forests; Central Sierra Lower Montane Forests	Ponderosa pine forests, mixed conifer forest, Jeffrey pine, giant sequoia, white fir, red fir forests, lodgepole pine, montane chaparral, gray pine, montane hardwood forests, canyon live oak, interior live oak, black oak, shrubby tanoak, manzanita, chamise, ceanothus, and other shrubs.
Miles Fire	7/15/2018	9/3/2018	54,334	Jackson, OR; Douglas, OR	Umpqua National Forest, Crater Lake National Park, Rogue River National Forest	42.848, -122.711	Western Cascades Lowlands and Valleys; Western Cascades Montane Highlands; High Southern Cascades Montane Forest; Cascades Subalpine/Alpine; Southern Cascades	Douglas-fir, western hemlock, western redcedar, bigleaf maple, red alder, vine maple, salal, rhododendron, Oregon grape, huckleberry, thimbleberry, swordfern, oxalis, hazel, and blackberry; Pacific silver fir, mountain hemlock, noble fir, Pacific yew; lodgepole pine; grand fir, white fir, Shasta red fir, woodrush, prince’s pine, lupine, sidebells shinleaf, whitebark pine, Shasta buckwheat, Newberry knotweed, and Brewer’s sedge; Herbaceous and shrubby subalpine meadow vegetation, subalpine fir, Holm’s sedge, black alpine sedge, tufted hairgrass, and alpine aster; ponderosa pine, incense cedar, sugar pine, snowberry, twinflower, serviceberry, golden chinkapin, and oceanspray.

Table S1 (Continued)

Fire name	Start day	End day	Burned area (acres)	County	Location	Lat/Lon	Ecosystem	Corresponding Vegetation
Garner Complex Fire	7/15/2018	8/14/2018	8,886	Josephine, OR	From west of Grants Pass toward northeast	42.556, -123.380	Oak Savanna Foothills; Rogue/Illinois Valleys; Inland Siskiyous; Serpentine Siskiyous	Oregon white oak, California black oak woodlands, madrone, ponderosa pine, grassland savanna, Douglas-fir, incense cedar, oceanspray, poison oak, snowberry, Idaho fescue, California brome, roughstalk bluegrass, ceanothus; grassland, California fescue, serviceberry, willow and cottonwood; Oregon grape; Jeffrey pine, tanoak, chaparral, manzanita, and Lemmon needlegrass
Timber Crater 6	7/15/2018	8/16/2018	3,126	Klamath, OR	20 miles SW of Chemult, OR, and on the northeast of Crater Lake	43.073, -112.039	Pumice Plateau; High Southern Cascades Montane Forest; Pumice Plateau Basins	Lodgepole pine, ponderosa pine, white fir, antelope bitterbrush, Idaho fescue, mountain alder, stream dogwood, willow, quaking aspen, mountain hemlock, lodgepole pine, Pacific silver fir, grand fir, white fir, Shasta red fir, woodrush, prince's pine, lupine, sidebells shinleaf, white bark pine, Shasta buckwheat, Newberry knotweed, Brewer's sedge, aspen, and wetland vegetation
Taylor Creek Fire	7/15/2018	10/1/2018	52,839	Josephine, OR	the west of Merlin	42.528, -123.571	Rogue/Illinois Valleys; Oak Savanna Foothills; Serpentine Siskiyous; Inland Siskiyous	Oregon white oak, madrone, California black oak, ponderosa pine, grasslands, California fescue, snowberry, serviceberry, willow and cottonwood; madrone, grassland savanna, Douglas-fir, incense cedar, oceanspray, poison oak, Idaho fescue, California brome, roughstalk bluegrass, and ceanothus; Jeffrey pine, tanoak, chaparral composed of manzanita, ceanothus, and Lemmon needlegrass; Oregon grape, and poison oak.
Natchez Fire	7/15/2018	10/30/2018	38,134	Siskiyou, CA	Southeast of Cave Junction, OR	41.951, -123.546	Border High-Siskiyous; Western Klamath Low Elevation Forests; Serpentine Siskiyous; Inland Siskiyous	White fir forests, red fir forests, Jeffrey pine, Douglas-fir, Port Orford cedar, tanoak, canyon live oak, red and white alder, mixed oak, incense cedar, manzanita, ceanothus, Idaho fescue, Lemmon needlegrass, ponderosa pine, Oregon white oak, California black oak, madrone, serviceberry, snowberry, Oregon grape, California fescue, and poison oak

Table S1 (Continued)

Fire name	Start day	End day	Burned area (acres)	County	Location	Lat/Lon	Ecosystem	Corresponding Vegetation
Klondike Fire	7/15/2018	11/16/2018	175,258	Josephine, OR	Siskiyou National Forest, southwest of Grants Pass, 9 miles west of Selma	42.369, -123.860	Serpentine Siskiyous; Inland Siskiyous; Oak Savanna Foothills; Rogue/Illinois Valleys	Jeffrey pine, tanoak, incense cedar, manzanita, ceanothus, Idaho fescue, Lemmon needlegrass, ponderosa pine, Oregon white oak, California black oak, madrone, serviceberry, snowberry, Oregon grape, California fescue, poison oak, Douglas-fir Forest, grassland savanna, oceanspray, California brome, roughstalk bluegrass, grasslands, willow, and cottonwood
Carr Fire	7/23/2018	8/30/2018	92,936	Shasta, CA; Trinity, CA	Hwy 299 and Carr Powerhouse Rd, Whiskeytown	40.654, -122.623	Eastern Klamath Low Elevation Forests; Eastern Klamath Montane Forests; Foothill Ridges and Valleys; Tehama Terraces	Douglas-fir, ponderosa pine, canyon live oak, knobcone pine, chaparral, chamise, deer brush, manzanita, cottonwoods, white alder, willows, white fir, incense cedar, sugar pine, mountain dogwood, Shasta fir, red fir, black oak, conifers, huckleberry oak, purple needlegrass, blue oak, foothill pine, needlegrass
Perry Fire	7/27/2018	8/5/2018	51,400	Washoe, NV	Southwest of Pyramid Lake, NV		Sierra Nevada-Infl uenced Semiarid Hills and Basins; Sierra Nevada-Infl uenced Ranges; Lahontan Sagebrush Slopes; Lahontan Salt Shrub Basin	Wyoming big sagebrush, antelope bitterbrush, desert peach, low sagebrush, Lahontan sagebrush, Douglas rabbitbrush, Nevada ephedra, bottlebrush squirreltail, desert needlegrass, Thurber needlegrass, Indian ricegrass, cheatgrass, bluebunch wheatgrass, Sandberg bluegrass, juniper, Riparian cottonwood woodland, buffaloberry, Singleleaf pinyon, Utah juniper, mountain big sagebrush, desert bitterbrush, green ephedra, currant, mountain brush, white fir, ponderosa pine, lodgepole pine, Jeffrey pine, western white pine, whitebark pine, littleleaf horsebrush, rabbitbrush, grasses, Shadscale, bud sagebrush, Bailey greasewood, Shockley desert thorn, Nevada eohedra, black greasewood, inland saltgrass, alkali sacaton, seepweed,indigobush, four-wing saltbush, dune horsebrush, Frémont cottonwood, coyote willow, silver buffaloberry, and Russian olive

Table S1 (Continued)

Fire name	Start day	End day	Burned area (acres)	County	Location	Lat/Lon	Ecosystem	Corresponding Vegetation
Whaleback Fire	7/27/2018	8/7/2018	18,703	Lassen, CA	Off Whaleback Mountain and Antelope Valley Road, West of Spaulding, west of Eagle Lake	40.633, -120.868	Modoc/Lassen Juniper-Shrub Hills and Mountains; California Cascades Eastside Conifer Forest; Low Southern Cascades Mixed Conifer Forest	Western juniper, big sagebrush, perennial bunchgrasses, Jeffrey pine, ponderosa pine, white fir, sagebrush, mixed conifer, western hemlock, western redcedar, incense cedar, Shasta red fir, manzanita, ceanothus, Curl-leaf mountain-mahogany, and antelope bitterbrush
Mendocino Complex Fire	7/27/2018	9/18/2018	459,123	Mendocino, CA; Lake, CA; Colusa, CA; Glenn, CA	Off Highway 20 near Potter Valley, northeast of Ukiah, north and southwest of Clear Lake	39.243, -123.103	Outer North Coast Ranges; North Coast Range Eastern Slopes; Clear Lake Hills and Valleys; Mayacmas Mountains; High North Coast Ranges	Tanoak, Oregon white oak, needlegrass grasslands, redwood, leather oak, chamise, mixed conifer, McNab or Sargent cypress, knobcone pine, blue oak woodland, cropland, orchards, vineyards, chaparral, annual grassland, montane hardwood, Douglas-fir forests, white fir forest, and red fir
Donnell Fire	8/1/2018	11/28/2018	36,450	Tuolumne, CA	Near Hwy 108, Donnell Lake area in the Carson Iceburg Wilderness	38.349, -119.929	Northern Sierra Upper Montane Forests; Northern Sierra Subalpine Forests; Central Sierra Mid-Montane Forests; Southern Sierra Subalpine Forests	Conifers, red fir, white fir, Jeffrey pine, sugar pine, incense cedar, lodgepole pine, quaking aspen, montane chaparral, whitebark pine, western white pine, mountain hemlock, mixed conifer forest, Douglas-fir, ponderosa pine, canyon live oak, black oak, foxtail pine, and limber pine
Wagner Fire	8/4/2018	8/7/2018	22	Mariposa, CA	Texas Hill Rd and Bondurant Mine Rd, east of Coulterville	37.699, -120.054	Central Sierra Lower Montane Forests; Southern Sierra Mid-Montane Forests	Gray pine, mixed conifer forest, Montane hardwood forests, canyon live oak, interior live oak, black oak, shrubby tanoak, mixed chaparral, manzanita, chamise, ceanothus, other shrubs, ponderosa pine forests, Jeffrey pine, and giant sequoia
Hat Fire	8/9/2018	8/16/2018	1900	Shasta, CA	Hwy 299 east and Fish Hatchery rd, northeast of Burney in the Fall River Mills area	40.993, -121.522	California Cascades Eastside Conifer Forest	Ponderosa pine, Jeffrey pine, western juniper, sagebrush
Hirz Fire	8/9/2018	9/12/2018	46150	Shasta, CA	Off Gilman and Moore Creek Campground, east of Lakehead	40.896, -122.219	Eastern Klamath Low Elevation Forests	Douglas-fir, ponderosa pine, canyon live oak, knobcone pine, chaparral of chamise, deer brush, manzanita, streams, cottonwoods, white alder, and willows

Table S1 (Continued)

Fire name	Start day	End day	Burned area (acres)	County	Location	Lat/Lon	Ecosystem	Corresponding Vegetation
Blue Jay Fire	7/24/2020	11/20/2020	6,922	Mariposa, CA; Tuolumne, CA	Near Yosemite Creek Campground		Southern Sierra Upper Montane Forests; Southern Sierra Subalpine Forests; Sierran Alpine	Mixed conifer, white fir, red fir forests, Jeffrey pine, lodgepole pine, montane chaparral, western white pine, mountain hemlock, foxtail pine, limber pine, Cushion plants, tufted grasses, alpine willows, sedges, various shrubs, herbaceous plants, krummholz forms of high-elevation conifers, and whitebark pine
Red Salmon Complex Fire	7/27/2020	11/17/2020	144,698	Humboldt, CA; Siskiyou, CA; Trinity, CA	Trinity Alps Wilderness Area, 14 miles northeast of Willow Creek, CA	41.185, -123.433	Marble/Salmon Mountains-Trinity Alps; Western Klamath Low Elevation Forests	Douglas-fir, white fir, red fir, Port Orford cedar, tanoak, canyon live oak, red and white alder, mixed oak
Wolf Fire	8/11/2020	11/19/2020	2,057	Tuolumne, CA	East of White Wolf (Yosemite)	34.609, -119.365	Southern Sierra Mid-Montane Forests; Southern Sierra Upper Montane Forests; Southern Sierra Subalpine Forests	Ponderosa pine forests, mixed conifer forest, Jeffrey pine, giant sequoia, white fir, red fir forests, lodgepole pine, montane chaparral, whitebark pine, western white pine, mountain hemlock, foxtail pine, and limber pine
Lake Fire	8/12/2020	9/28/2020	31,089	Los Angeles, CA	Lake Hughes Rd and Prospect Rd, southwest Lake Hughes	34.679, -118.452	Southern California Lower Montane Shrub and Woodland	Chaparral, bigcone Douglas-fir, canyon live oak, coastal sage scrub
Ranch 2 Fire	8/13/2020	10/5/2020	4,237	Los Angeles, CA	North San Gabriel Canyon Rd and Ranch Rd, San Gabriel Canyon	34.160, -117.910	Los Angeles Plain	California sagebrush, California buckwheat, coast live oak, chamise chaparral, and annual grasslands
Loyalton Fire	8/14/2020	9/14/2020	47,029	Lassen, CA; Plumas, CA; Sierra, CA; Washoe, NV	Mount Ina Coolbrith East of Loyalton in Sierra County	39.702, -120.143	Sierra Nevada-Infl uenced Semiarid Hills and Basins; Northeastern Sierra Mixed Conifer-Pine Forests; Sierra Nevada-Influenced Ranges; Sierra Valley	Wyoming big sagebrush, antelope bitterbrush, desert peach, low sagebrush, Lahontan sagebrush, Douglas rabbitbrush, Nevada ephedra, bottlebrush squirreltail, desert needlegrass, Thurber needlegrass, Indian ricegrass, cheatgrass, bluebunch wheatgrass, Sandberg bluegrass, juniper, Riparian cottonwood woodland, buffaloberry, conifers, Jeffrey pine, ponderosa pine, sugar pine, incense cedar, California white fir, sagebrush, fire-maintained chaparral, snowbrush, and manzanita; pinyon–juniper woodland, lodgepole pine, western white pine, grassland, cattail, and bulrush

Table S1 (Continued)

Fire name	Start day	End day	Burned area (acres)	County	Location	Lat/Lon	Ecosystem	Corresponding Vegetation
Dome Fire	8/15/2020	8/24/2020	43,273	San Bernardino, CA	In the Mojave National Preserve	35.301, -115.598	Eastern Mojave Basins; Eastern Mojave Low Ranges and Arid Footslopes	Mesquite, acacia, smoke tree, Creosotebush, white bursage, galleta grass, Desert willow, coyote willow; blackbrush, Mojave Desert forbs, shrubs, succulent species including Joshua tree, yucca species, and cacti
Elk Fire	8/16/2020	8/17/2020	727	Glenn, CA	West of Willows	39.524, -122.427	North Valley Alluvium	Annual grasslands, blue oaks, and valley oaks
Crane Fire	8/16/2020	8/31/2020	2,993	Lake, OR	On Crane Mountain, near Crane Creek, 10 miles southeast of Lakeview	42.094, -120.269	Fremont Pine/Fir Forest	Ponderosa pine and western juniper, white fir, whitebark pine, lodgepole pine, snowberry, heartleaf arnica, Wheeler bluegrass, Antelope bitterbrush, and longstolon sedge
River Fire	8/16/2020	9/4/2020	48,088	Monterey, CA	Pine Canyon and River Rd, east of Salinas	36.602, -121.621	Northern Santa Lucia Range; Monterey Bay Plains and Terraces	Coast live oak, California sagebrush-black sage, chamise and live oak shrublands, Douglas-fir, tanoak, redwood; California oatgrass, herbaceous communities, coastal scrub, sage, pickleweed, cropland, artichokes, strawberries, and lettuce
CZU Lightning Complex Fire	8/16/2020	9/22/2020	86,509	San Mateo, CA; Santa Cruz, CA	Various locations across San Mateo and Santa Cruz Counties	37.171, -122.223	Santa Cruz Mountains; San Mateo Coastal Hills	Redwood, Douglas-fir, tanoak, coast live oak, California bay, chaparral, coastal scrub, Santa Cruz cypress, Shreve oak, and cropland
SCU Lightning Complex Fire	8/16/2020	10/1/2020	396,624	Santa Clara, CA; Alameda, CA; Contra Costa, CA; San Joaquin, CA; Merced, CA; Stanislaus, CA	Multiple locations throughout Santa Clara County, Alameda County, Contra Costa County, San Joaquin County, Merced and Stanislaus County	37.439, -121.304	Diablo Range; East Bay Hills/Western Diablo Range; Eastern Hills; Westside Alluvial Fans and Terraces; Livermore Hills and Valleys; Delta; San Joaquin Basin	Blue oak woodlands, savannas, chamise, leather oak, mixed chaparral shrublands, sargent cypress, black oak, mixed conifers,Jeffrey pine, coast live oak, valley oak, chamise, eucalyptus, flammable exotic vegetation, needlegrass, annual grasslands, agricultural land, perennial, bladderpod, ephedra shrublands, coastal live oak, corn, alfalfa, wheat, aquatic plants, tules, cattails, rushes, cropland, hay/pasture land, alfalfa, tomatoes, emergent aquatics, cottonwood, willow, saltgrass, alkali sacaton, creeping wildrye, and allscale

Table S1 (Continued)

Fire name	Start day	End day	Burned area (acres)	County	Location	Lat/Lon	Ecosystem	Corresponding Vegetation
Beachie Creek Fire	8/16/2020	10/28/2020	193,573	Clackamas, OR; Linn, OR; Marion, OR	In the Opal Creek Wilderness about six miles northwest of Detroit Lake in Oregon, about 38 miles east of Salem, approximately two miles south of Jawbone Flats on the Willamette National Forest		Valley Foothills; Western Cascades Lowlands and Valleys; Western Cascades Montane Highlands	Oak savanna, prairies, California oatgrass, fescue, blue wildrye, brodiaea, prairie forbs, Douglas-fir forests, sword fern, oceanspray, hazel, baldhip rose, poison oak, and alien Himalayan blackberry; western hemlock, western redcedar, bigleaf maple, red alder, vine maple, salal, rhododendron, Oregon grape, huckleberry, thimbleberry, oxalis, hazel, Pacific silver fir, mountain hemlock, noble fir, bigleaf maple, and Pacific yew.
August Complex Fire	8/16/2020	11/12/2020	1,032,648	Glenn, CA; Lake, CA; Mendocino, CA; Tehama, CA; Trinity, CA; Shasta, CA	Mendocino National Forest, with portions spilling over to the Shasta-Trinity National Forest and Six Rivers National Forest in the north, as well as private land surrounding the forests.	39.765, -122.673	High North Coast Ranges; Outer North Coast Ranges; North Coast Range Eastern Slopes; Foothill Ridges and Valleys; Eastern Klamath Montane Forests;	Mixed conifer, Douglas-fir forests, tanoak, white fir forest, red fir, Oregon white oak, needlegrass grasslands, redwood, leather oak, chamise, mixed conifer, McNab or Sargent cypress, knobcone pine, blue oak woodland, purple needlegrass, foothill pine, incense cedar, ponderosa pine, sugar pine, mountain dogwood, Shasta fir, black oak, canyon live oaks, huckleberry oak and chaparral
Lionshead Fire	8/16/2020	11/13/2020	204,469	Jefferson, OR; Linn, OR; Marion, OR; Wasco, OR	14 miles west of the Warm Springs community		Western Cascades Lowlands and Valleys; Western Cascades Montane Highlands; Cascades Subalpine/Alpine; Ponderosa Pine/Bitterbrush Woodland	Douglas-fir, western hemlock, western redcedar, bigleaf maple, red alder, vine maple, salal, rhododendron, Oregon grape, huckleberry, thimbleberry, swordfern, oxalis, hazel, and blackberry; Pacific silver fir, mountain hemlock, noble fir, Pacific yew; Herbaceous and shrubby subalpine meadow vegetation, whitebark pine, and subalpine fir, Brewer's sedge, Holm's sedge, black alpine sedge, tufted hairgrass, and alpine aster; Ponderosa pine, antelope bitterbrush, greenleaf manzanita, snowberry, mountain alder, stream dogwood, willows, and sedges.
Jones Fire	8/17/2020	8/28/2020	705	Nevada, CA	Jones Bar Rd, Yuba River Drainage, northwest of Nevada City	39.292, -121.100	Northern Sierra Lower Montane Forests; Northern Sierra Mid-Montane Forests; Northern Sierran Foothills	Montane hardwood, montane hardwood-conifer, mixed conifer forests, Douglas-fir, canyon live oak, interior live oak, black oak, tanoak, white fir, ponderosa pine, Jeffrey pine, needlegrass, annual grasslands, chamise, manzanita, ceanothus, blue oak, and foothill pine

Table S1 (Continued)

Fire name	Start day	End day	Burned area (acres)	County	Location	Lat/Lon	Ecosystem	Corresponding Vegetation
Holser Fire	8/17/2020	9/6/2020	3,000	Ventura, CA	Holser Canyon Rd and Piru Canyon Rd, south of Lake Piru	34.439, -118.759	Venturan-Angeleno Coastal Hills; Oxnard Plain and Valleys	Annual grassland, California sagebrush, California buckwheat, mixed sage scrub, chamise chaparral, mixed chaparral, and coast live oak; brome and needlegrass grasslands, cropland, citrus orchards
Sheep Fire	8/17/2020	9/9/2020	29,570	Plumas, CA	Hennignan's Sheep Camp, PNF; Gold Run Road, Southwest of Susanville in Plumas County.	40.274, -120.757	Northeastern Sierra Mixed Conifer-Pine Forests; Northern Sierra Upper Montane Forests; Sierra Nevada-Infl uenced Semiarid Hills and Basins; Northern Sierra Mid-Montane Forests	Mix of conifers, Jeffrey pine, ponderosa pine, sugar pine, incense cedar, California white fir, sagebrush, antelope bitterbrush, fire-maintained chaparral, snowbrush, manzanita, red fir, white fir, lodgepole pine, quaking aspen, montane chaparral, Wyoming big sagebrush, desert peach, low sagebrush, Lahontan sagebrush, Douglas rabbitbrush, Nevada ephedra, bottlebrush squirreltail, desert needlegrass, Thurber needlegrass, Indian ricegrass, cheatgrass, bluebunch wheatgrass, Sandberg bluegrass, juniper, Riparian cottonwood woodland, buffaloberry, Douglas-fir, black oak, tanoaks, and canyon live oak
LNU Lightning Complex Fire	8/17/2020	10/2/2020	363,220	Lake, CA; Napa, CA; Sonoma, CA; Solano, CA; Yolo, CA	Arround Lake Berryessa	38.482, -122.149	Foothill Ridges and Valleys; North Coast Range Eastern Slopes; Western Valley Foothills/Dunnigan Hills; Napa-Sonoma-Lake Volcanic Highlands; Yolo Alluvial Fans	Purple needlegrass, chamise, foothill pine, leather oak, chamise, mixed conifer, McNab or Sargent cypress, knobcone pine, blue oak woodland, needlegrass grasslands, mixed chaparral, mixed hardwoods, coast live oak, madrone, California bay, Douglas-fir, Douglas pine, cypress, grasslands, valley oak, cottonwoods, willows, cropland, pastureland, alfalfa, winter wheat, sunflowers, corn, tomatoes, strawberries, stonefruit, walnut, and almond orchards
North Complex Fire	8/17/2020	12/3/2020	318,935	Plumas,CA; Butte, CA	in the Plumas National Forest, east of Lake Oroville	40.091, -120.931	Northern Sierra Lower Montane Forests; Northern Sierra Mid-Montane Forests; Northern Sierran Foothills; Northern Sierra Upper Montane Forests	Montane hardwood, montane hardwood-conifer, mixed conifer forests, Douglas-fir, canyon live oak, interior live oak, black oak, tanoak, white fir, ponderosa pine, Jeffrey pine, needlegrass grassland, annual grassland, chamise, manzanita, ceanothus, blue oak, foothill pine, red fir, white fir, sugar pine, incense cedar, lodgepole pine, quaking aspen, and montane chaparral

Table S1 (Continued)

Fire name	Start day	End day	Burned area (acres)	County	Location	Lat/Lon	Ecosystem	Corresponding Vegetation
Salt Fire	8/18/2020	8/24/2020	1,789	Calaveras, CA	Salt Springs Valley Reservoir	38.028, -120.763	Northern Sierran Foothills	Needlegrass, annual grasslands, chamise, manzanita, interior live oak, ceanothus, blue oak, and foothill pine
Carmel Fire	8/18/2020	9/4/2020	6,905	Monterey, CA	Cachagua Rd and Carmel Valley Rd, South of Carmel	36.446, -121.682	Northern Santa Lucia Range	Coast live oak, California sagebrush-black sage, canyon live oak, chamise and live oak shrublands, Douglas-fir, tanoak, and redwood
W-5 Cold Spring Fire	8/18/2020	9/14/2020	84,817	Lassen, CA	Cold Spring Road, West of Cold Spring Mountain	41.028, -120.281	Modoc/Lassen Juniper-Shrub Hills and Mountains; Warner Mountains; High Elevation Warner Mountains; High Lava Plains; Pluvial Lake Basins	Western juniper, big sagebrush, perennial bunchgrasses, Jeffrey pine, ponderosa pine, white fir, low sagebrush, perennial bunchgrasses, Washoe pine, aspen, lodgepole pine, whitebark pine, Drought-tolerant alpine cushion plants, bluebunch wheatgrass, Wyoming big sagebrush, cheatgrass, greasewood, inland saltgrass, seepweed, basin big sagebrush, and associated grasses
Woodward Fire	8/18/2020	10/1/2020	4,929	Marin, CA	Woodward Valley Trail, East of Olema	38.018, -122.836	Marin Hills; Coastal Franciscan Redwood Forest	Coast live oak, annual grasslands, coastal scrub, tanoak, Douglas-fir, multi-story canopy of redwood, bigleaf maple, evergreen shrubs, various grasses, and grassland savannas
Dolan Fire	8/18/2020	12/31/2020	128,050	Monterey, CA	In the Big Sur region and other parts of the Santa Lucia Mountain range	36.123, -121.602	Santa Lucia Coastal Forest and Woodland; Northern Santa Lucia Range	Douglas-fir, tanoak, redwoods, shrub; Coast live oak, California sagebrush-black sage, Canyon live oak, chamise
SQF Lightning Complex Fire	8/19/2020	1/5/2021	174,178	Tulare, CA	25 miles north of Kernville, CA	36.255, -118.497	Southern Sierran Foothills; Southern Hardpan Terraces	Blue oak, needlegrass, annual grasslands, chamise, ceanothus, mixed oaks, ceanothus shrublands, blue oak savannas, and foothill pine
Moc Fire	8/20/2020	8/30/2020	2,857	Tuolumne, CA	West of Groveland; Hwy 49 and Hwy 120, Moccasin	37.814, -120.312	Northern Sierran Foothills; Central Sierra Lower Montane Forests	Needlegrass grassland, annual grassland, chamise, manzanita, interior live oak, ceanothus, blue oak, foothill pine, ponderosa pine, gray pine, mixed conifer forest, Montane hardwood forests, canyon live oak, black oak, shrubby tanoak, mixed chaparral, and other shrubs

Table S1 (Continued)

Fire name	Start day	End day	Burned area (acres)	County	Location	Lat/Lon	Ecosystem	Corresponding Vegetation
Slink Fire	8/29/2020	11/8/2020	26,759	Alpine, CA; Mono, CA	Slinkard Valley, west of Coleville	38.568, -119.568	Northeastern Sierra Mixed Conifer-Pine Forests; Sierran Alpine; Northern Sierra Subalpine Forests	Mid-elevation dry forests, mix of conifers, Jeffrey pine, ponderosa pine, sugar pine, incense cedar, California white fir, sagebrush, antelope bitterbrush, fire-maintained chaparral, snowbrush, manzanita, Cushion plants, tufted grasses, alpine willows, sedges, various shrubs, herbaceous plants, krummholz forms of high-elevation conifers, whitebark pine, lodgepole pine, western white pine, and mountain hemlock
Creek Fire	9/4/2020	12/24/2020	379,895	Fresno, CA; Madera, CA	Both sides of the San Joaquin River near Mammoth Pool, Shaver Lake, Big Creek and Huntington Lake	37.1915, -119.261	Southern Sierra Mid-Montane Forests; Southern Sierra Lower Montane Forest and Woodland; Southern Sierra Upper Montane Forests; Southern Sierra Subalpine Forests; Sierran Alpine	Ponderosa pine forests, mixed conifer forest, Jeffrey pine, giant sequoia, oak, chaparral, white fir, red fir forests, lodgepole pine, montane chaparral, western white pine, mountain hemlock, foxtail pine, limber pine, Cushion plants, tufted grasses, alpine willows, sedges, various shrubs, herbaceous plants, krummholz forms of high-elevation conifers, and whitebark pine
El Dorado Fire	9/5/2020	11/16/2020	22,744	San Bernardino, CA; Riverside, CA	In El Dorado Ranch Park, and quickly spread to the San Gorgonio Wilderness Area of the San Bernardino National Forest	34.058, -116.989	Inland Hills; Inland Valleys; Southern California Lower Montane Shrub and Woodland; Southern California Montane Conifer Forest	Sage scrub, grassland, and chaparral; riparian woodlands, cropland; mixed evergreen woodland, bigcone Douglas-fir, canyon live oak, shrubs such as California buckwheat, are ubiquitous; mixed coniferous forest with ponderosa pine, Jeffrey pine, sugar pine, white fir, incense cedar, hardwoods such as black oak
Bobcat Fire	9/6/2020	12/18/2020	115,997	Los Angeles, CA	In the central San Gabriel Mountains, in and around the Angeles National Forest	34.241, -117.868	Southern California Lower Montane Shrub and Woodland	Chaparral, mixed evergreen woodland, bigcone Douglas-fir, canyon live oak, coastal sage scrub

Table S1 (Continued)

Fire name	Start day	End day	Burned area (acres)	County	Location	Lat/Lon	Ecosystem	Corresponding Vegetation
Echo Mountain Complex Fire	9/7/2020	9/21/2020	2,552	Lincoln, OR	4 miles east of Lincoln City, Oregon	45.015, -123.903	Coastal Lowlands; Coastal Uplands; Volcanics	Sitka spruce, western hemlock, Douglas-fir canopy, salal, sword fern, vine maple, and Oregon grape, red alder, western redcedar, bigleaf maple, salmonberry; California bay-laurel, Baltic rush, Lyngby's sedge, tufted hairgrass, Pacific silverleaf, seaside arrowgrass, shore pine, sweet gale, and Hooker's willow, shore pine, rhododendron, evergreen blueberry shrub, dune wildrye, Chilean strawberry, and dune bentgrass; bigleaf maple, Grasslands; salal, oxalis, Roemer's fescue, thin bentgrass, California oatgrass, and diverse forbs.
Brattain Fire	9/7/2020	10/6/2020	50,951	Lake, OR	8 miles South of Paisley, Oregon	42.572, -120.558	Fremont Pine/Fir Forest; Pumice Plateau; High Desert Wetlands	Ponderosa pine, western juniper, white fir, whitebark pine, lodgepole pine, snowberry, heartleaf arnica, Wheeler bluegrass, antelope bitterbrush, longstolon sedge, Idaho fescue, mountain alder, stream dogwood, willows, quaking aspen, sedges, rushes, tufted hairgrass, black greasewood, mat muhley, creeping wildrye, bluegrass, basin big sagebrush, Wyoming big sagebrush, silver sagebrush, basin wildrye, Nevada bluegrass, bluebunch wheatgrass, Thurber needlegrass, and cheatgrass
242 Fire	9/7/2020	10/10/2020	14,473	Klamath, OR	Hwy 97 mp243 south to mp248, Hwy 422, Hwy 62 mp85-mp100, 1mi W/NW of Chiloquin	42.653, -121.869	Klamath/Goose Lake Basins; Pumice Plateau	Bluebunch wheatgrass, Idaho fescue, Antelope bitterbrush, mountain big sagebrush, low sagebrush, basin wildrye, basin big sagebrush, tules, cattails, and sedges; lodgepole pine, ponderosa pine, white fir, antelope bitterbrush, mountain alder, stream dogwood, willows, and quaking aspen
South Obenchain Fire	9/8/2020	10/3/2020	32,671	Jackson, OR	5 miles east of Eagle Point, Oregon	42.467, -122.683	Rogue/Illinois Valleys; Oak Savanna Foothills	Oregon white oak, madrone, California black oak, ponderosa pine, grasslands, California fescue, snowberry, serviceberry, willow, cottonwood, grassland savanna, incense cedar, oceanspray, poison oak, Idaho fescue, California brome, roughstalk bluegrass, and ceanothus

Table S1 (Continued)

Fire name	Start day	End day	Burned area (acres)	County	Location	Lat/Lon	Ecosystem	Corresponding Vegetation
Thielsen Fire	9/8/2020	11/16/2020	9,975	Douglas, OR	5 miles north of Crater Lake National Park and within 1 mile of Diamond Lake Resort	43.160, -122.070	High Southern Cascades Montane Forest	Mountain hemlock, lodgepole pine, Pacific silver fir,grand fir, white fir, Shasta red fir,woodrush, prince's pine, lupine, sidebells shinleaf,white bark pine,Shasta buckwheat, Newberry knotweed, and Brewer's sedge
Archie Creek Fire	9/8/2020	11/16/2020	131,542	Douglas, OR	Steamboat Springs on Hwy 138E to Glide, Oregon	43.334, -122.788	Western Cascades Lowlands and Valleys; Umpqua Interior Foothills	Western hemlock, western redcedar, bigleaf maple, red alder, vine maple, salal, rhododendron, Oregon grape, huckleberry, thimbleberry, swordfern, oxalis, hazel, blackberry, Oregon white oak, ponderosa pine, grand fir, madrone, tanoak, chinkapin, snowberry, poison oak, and oceanspray
Riverside Fire	9/8/2020	12/3/2020	138,054	Clackamas, OR	Near Riverside Campground		Western Cascades Lowlands and Valleys; Western Cascades Montane Highlands	Douglas-fir, western hemlock, western redcedar, bigleaf maple, red alder, vine maple, salal, rhododendron, Oregon grape, huckleberry, thimbleberry, swordfern, oxalis, hazel, and blackberry; Pacific silver fir, mountain hemlock, noble fir, Pacific yew.
Slater Fire	9/8/2020	11/16/2020	157,220	Josephine, OR	Community of Happy Camp and Surrounding Area	41.766, -123.375	Western Klamath Low Elevation Forests; Serpentine Siskiyous; Inland Siskiyous; Oak Savanna Foothills	Douglas-fir, Port Orford cedar, tanoak, canyon live oak, red alder, white alder, mixed oak, Jeffrey pine, incense cedar, manzanita, Idaho fescue, Lemmon needlegrass, ponderosa pine, Oregon white oak, California black oak, madrone, serviceberry, snowberry, Oregon grape, California fescue, poison oak, grassland savanna, oceanspray, California brome, roughstalk bluegrass, and ceanothus
Devil Fire	9/9/2020	11/16/2020	8,857	Josephine, OR	Community of Happy Camp and Surrounding Area	41.766, -123.375		

Table S1 (Continued)

Fire name	Start day	End day	Burned area (acres)	County	Location	Lat/Lon	Ecosystem	Corresponding Vegetation
Snow Fire	9/17/2020	10/6/2020	6,254	Riverside, CA	Snow Creek Road and Cottonwood Road, west of Palm Springs	33.888, -116.683	Upper Coachella Valley and Hills	California fan palms, agricultural lands
Zogg Fire	9/27/2020	10/13/2020	56,338	Shasta, CA; Tehama, CA	Zogg Mine Road and Jenny Bird Lane, North of Igo	40.539, -122.566	Foothill Ridges and Valleys; Tehama Terraces; Eastern Klamath Low Elevation Forests	purple needlegrass, blue oak, chamise, and foothill pine; Douglas-fir, ponderosa pine, canyon live oak, knobcone pine, chaparral of deer brush, manzanita. cottonwoods, white alder, and willows
Glass Fire	9/27/2020	10/20/2020	67,484	Napa, CA; Sonoma, CA	North Fork Crystal Springs Rd & Crystal Springs Rd	38.563, -122.497	North Coast Range Eastern Slopes; Napa-Sonoma-Russian River Valleys; Napa-Sonoma-Lake Volcanic Highlands	Chaparral, grassland, blue oak, mixed conifers, leather oak, foothill pine, knobcone pine; Grape vineyards, orchards, specialty crops; mixed hardwoods (including coast live oak, madrone, and California bay), Douglas-fir or pines, cypress.
Pony Fire	10/3/2020	10/4/2020	20	Sacramento, CA	Pony Brown Rd and Ione Rd, Sloughhouse	38.464, -121.039	Northern Terraces	Annual grass, forbs, blue oak woodlands

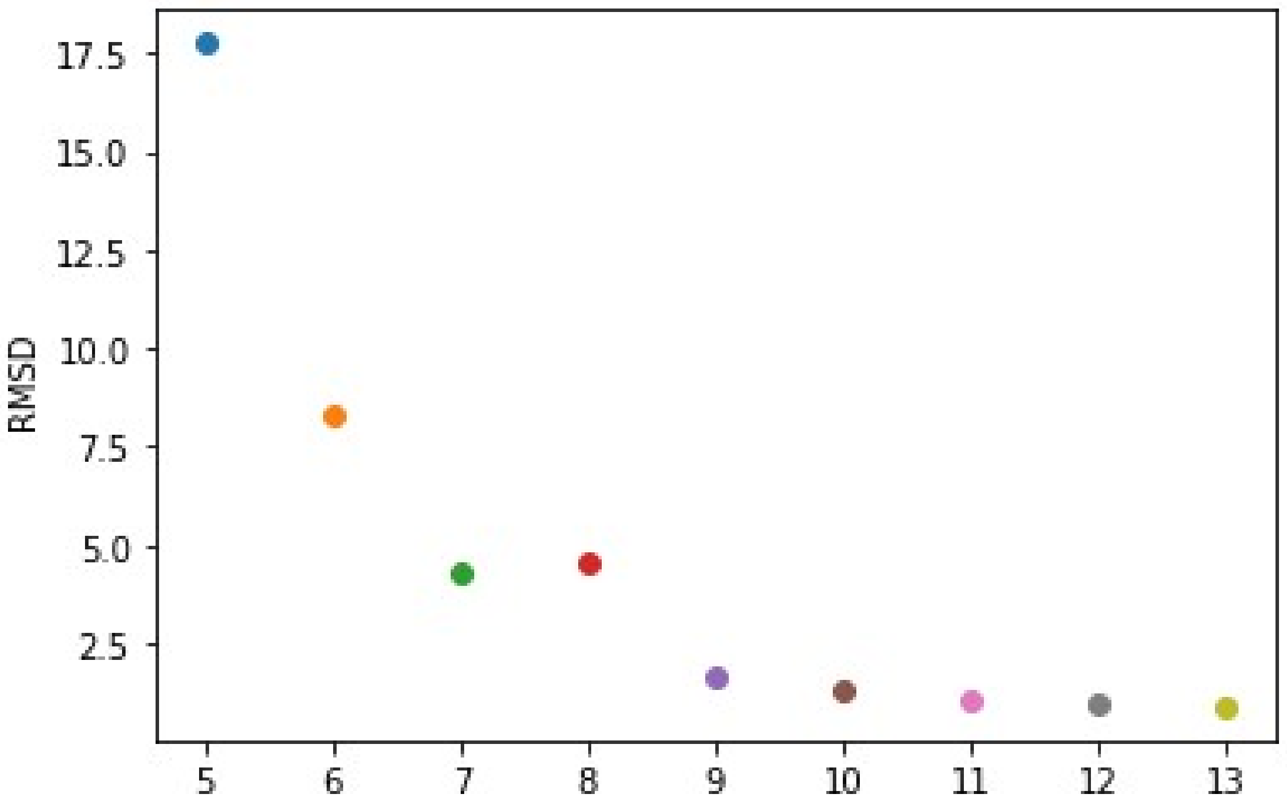

Figure S1. Root mean square deviation (RMSD) for factors from 5 to 13 on the x-axis.

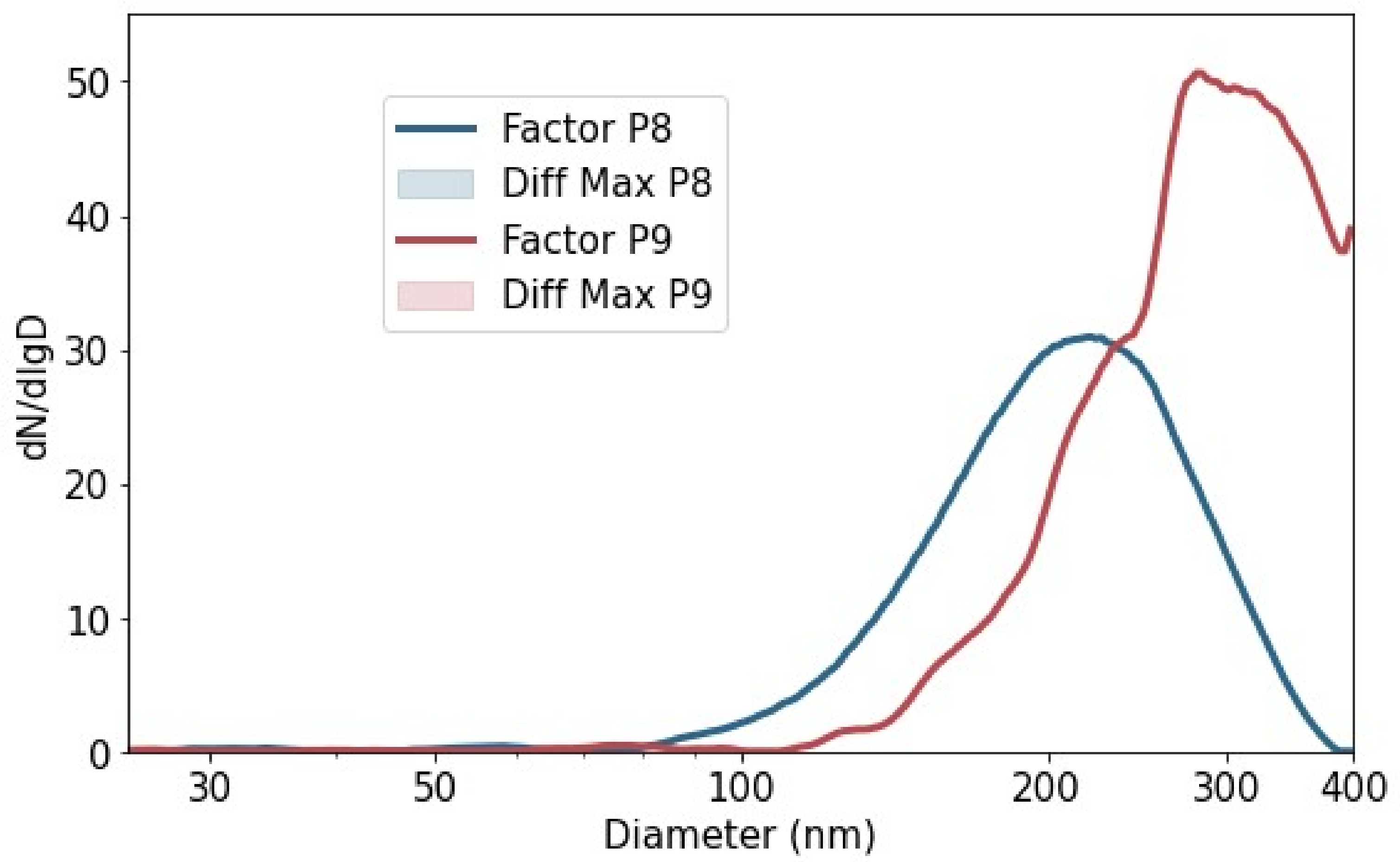

Figure S2. Sensitivity of the largest two PSD factors to 10% random perturbations of the input values. The results show the range of derived factors during 100 random simulations.

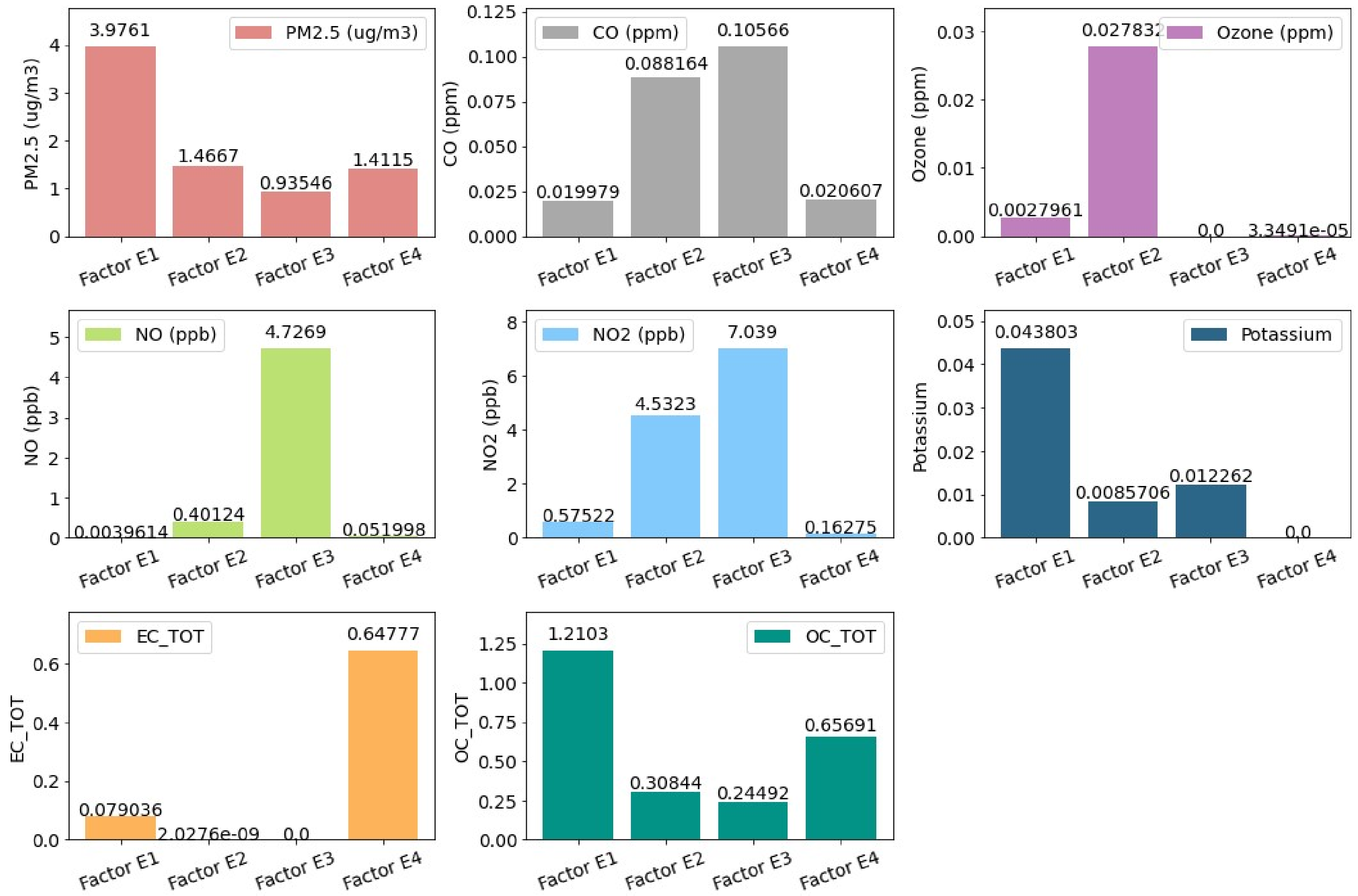

Figure S3. Detailed profile of each species from the EPA PMF.

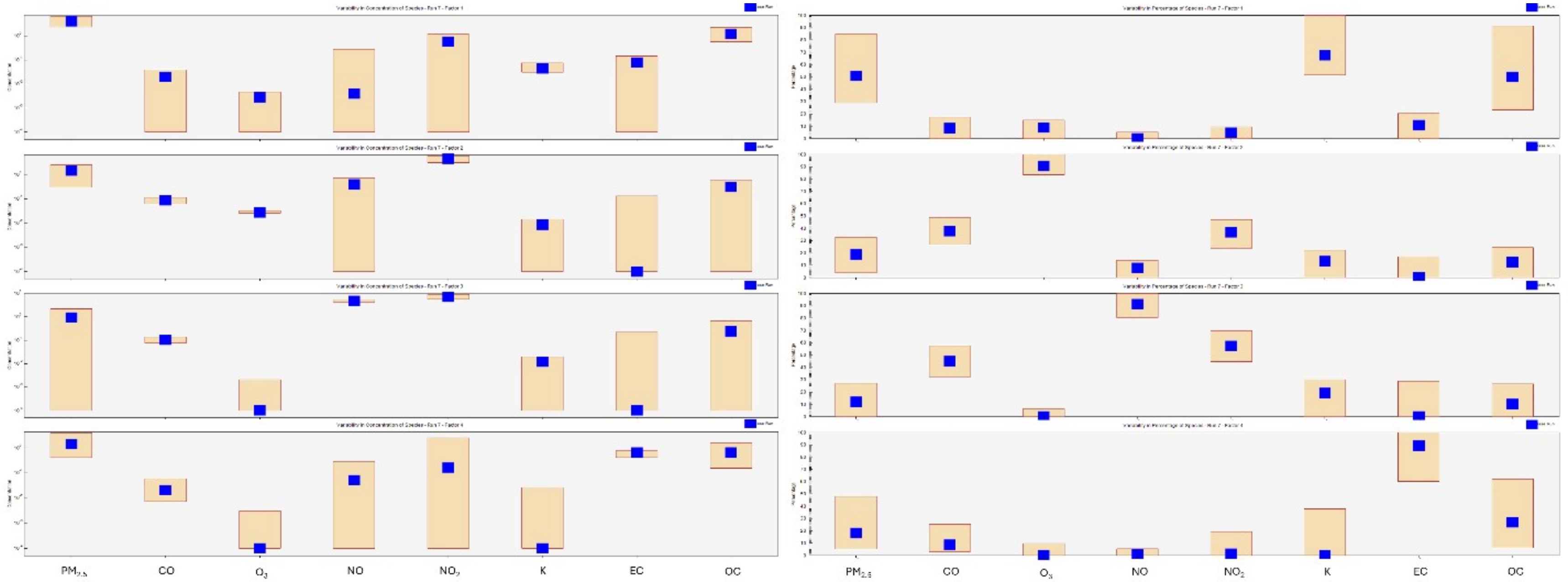

Figure S4. The variability of the selected solution from the EPA PMF was estimated through the BS-DISP error estimation analyses. The left panel shows the variability in concentration and percentage (right panel) of species from BS-DISP analysis. The blue squares represent profiles of the selected solution from the EPA PMF, and the yellow rectangles represent the ranges of variabilities.

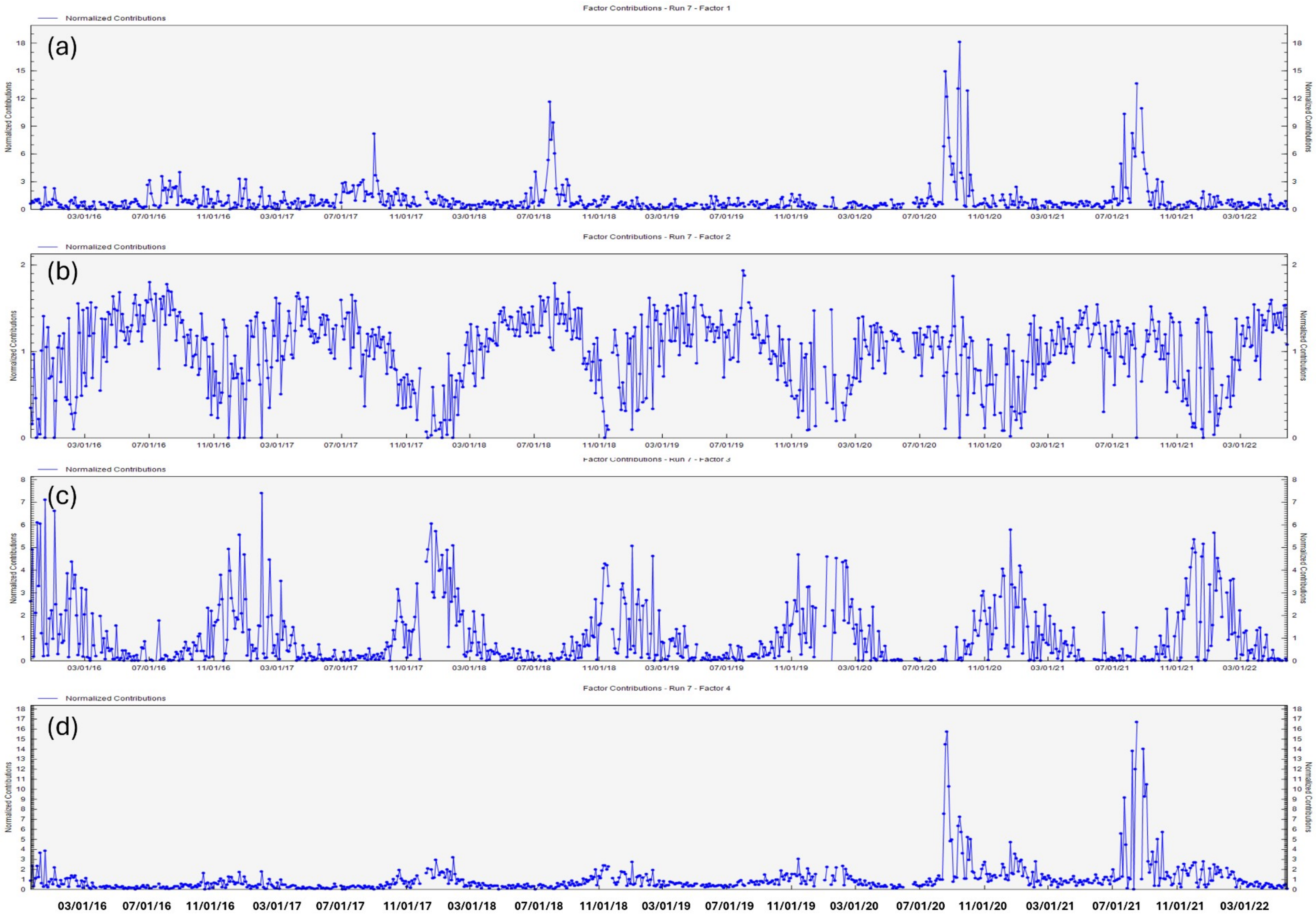

Figure S5. Contribution plot of all four factors from the EPA PMF. (a)-(d) are Factor E1-E4.

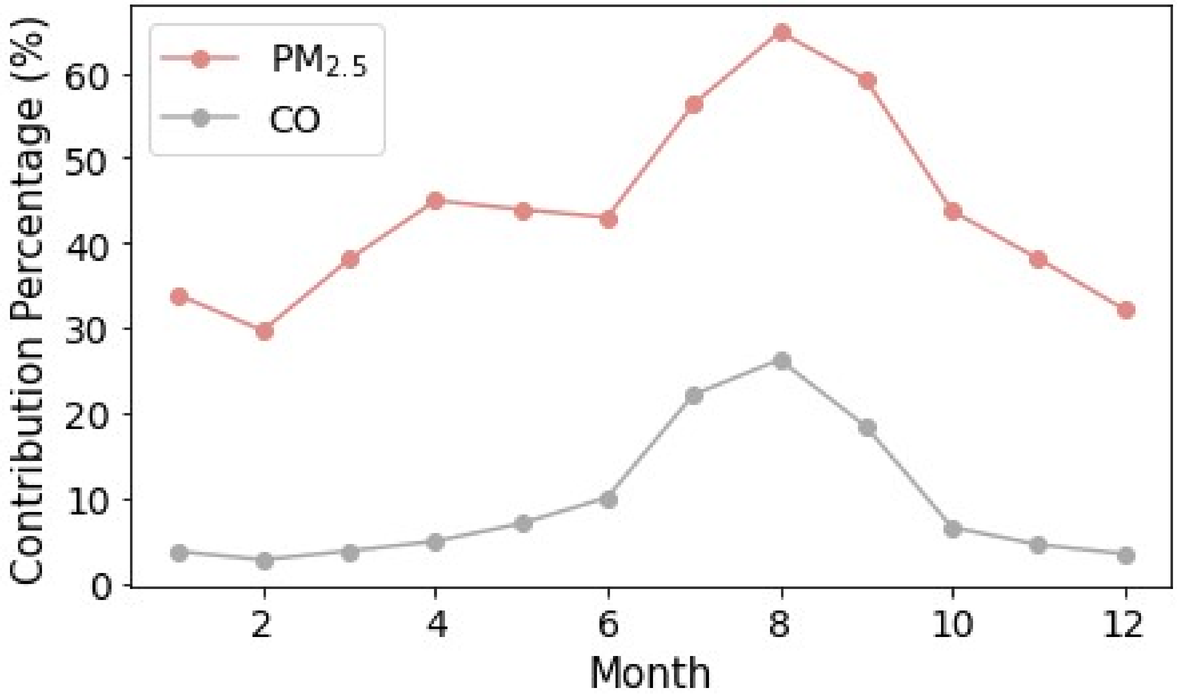

Figure S6. The monthly percentage of $PM_{2.5}$ and CO contribution for Factor E1 (wildfire-related factor).

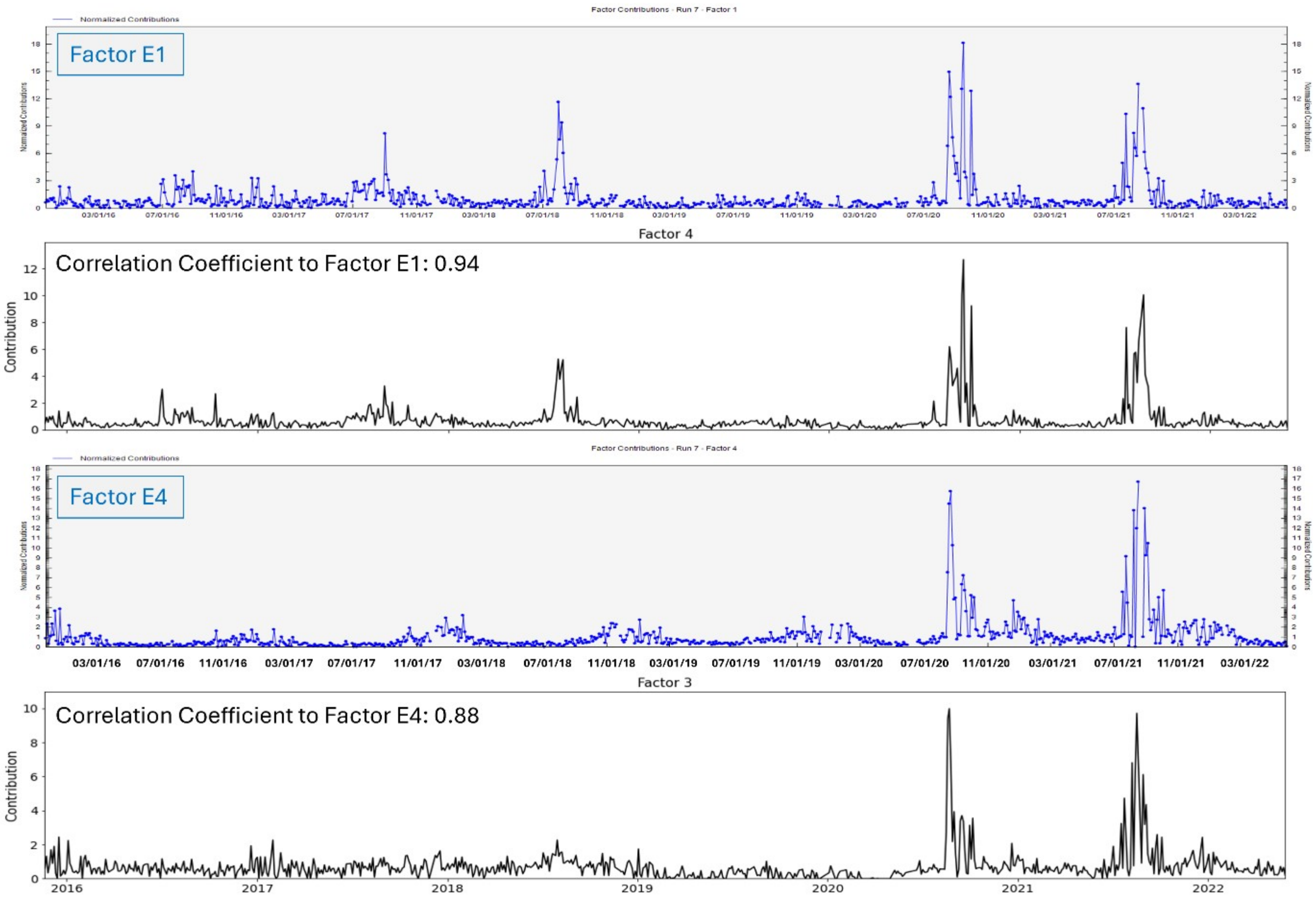

Figure S7. Comparison of fire-related factors from the EPA PMF and the Python PMF applied to the EPA data.
